# A “Weird” Mitochondrial Fatty Acid Oxidation as a Metabolic “Secret” of Cancer

**DOI:** 10.1155/2022/2339584

**Published:** 2022-02-08

**Authors:** Zhivko Zhelev, Ichio Aoki, Dessislava Lazarova, Tatyana Vlaykova, Tatsuya Higashi, Rumiana Bakalova

**Affiliations:** ^1^Department of Molecular Imaging and Theranostics, National Institutes for Quantum Science and Technology (QST), Chiba 263-8555, Japan; ^2^Faculty of Medicine, Trakia University, Stara Zagora 6000, Bulgaria; ^3^Institute of Biophysics and Biomedical Engineering, Bulgarian Academy of Sciences, Sofia 1113, Bulgaria; ^4^Faculty of Medicine, Sofia University “St. Kliment Ohridski”, Sofia 1407, Bulgaria

## Abstract

Cancer metabolism is an extensively studied field since the discovery of the Warburg effect about 100 years ago and continues to be increasingly intriguing and enigmatic so far. It has become clear that glycolysis is not the only abnormally activated metabolic pathway in the cancer cells, but the same is true for the fatty acid synthesis (FAS) and mevalonate pathway. In the last decade, a lot of data have been accumulated on the pronounced mitochondrial fatty acid oxidation (mFAO) in many types of cancer cells. In this article, we discuss how mFAO can escape normal regulation under certain conditions and be overactivated. Such abnormal activation of mitochondrial *β*-oxidation can also be combined with mutations in certain enzymes of the Krebs cycle that are common in cancer. If overactivated *β*-oxidation is combined with other common cancer conditions, such as dysfunctions in the electron transport complexes, and/or hypoxia, this may alter the redox state of the mitochondrial matrix. We propose the idea that the altered mitochondrial redox state and/or inhibited Krebs cycle at certain segments may link mitochondrial *β*-oxidation to the citrate-malate shuttle instead to the Krebs cycle. We call this abnormal metabolic condition “*β*-oxidation shuttle”. It is unconventional mFAO, a separate metabolic pathway, unexplored so far as a source of energy, as well as a source of cataplerosis, leading to biomass accumulation, accelerated oxygen consumption, and ultimately a source of proliferation. It is inefficient as an energy source and must consume significantly more oxygen per mole of ATP produced when combined with acetyl-CoA consuming pathways, such as the FAS and mevalonate pathway.

## 1. Introduction

The discussion of cancer cell metabolism always begins with Warburg's first unified theory of cancer about 100 years ago [1, 2]. The main postulate in this theory is the huge consumption of glucose in the tumor tissue, accompanied by the production of lactate, and this characteristic is attributed to the cancer cells themselves. Warburg wrote that this distinctive feature is the result of “irreversibly impaired respiration” and lists many respiratory inhibitors that cause cancer [2]. He concluded that respiration is impaired in these cells, but it should be noted that he never claimed that they cannot respire.

The debate over whether cancer cells have normal respiration or not arose after his article in *Science*, published in 1956 [2–4].This debate has long been in the background and even forgotten. Many studies have described cancer cells with well-expressed oxidative phosphorylation (OXPHOS) [5], as well as cancer cells that do not use glycolysis, for example, cancer cells using lactate from the surrounding noncancerous cells [6–8]. It has even been found that cancer cells can reverse the Warburg effect [9, 10]. However, findings that cancer cells can cope with respiration almost as normal cells and can consume lactate from surrounding noncancerous cells does not solve the problem, but raises more questions: “Why do tumors in general prefer to use the anaerobic part of glycolysis, when some cancer cells are characterized by good respiration, almost like normal cells?” The notion that different cancer cells have different metabolisms is gradually being adopted. Some cells in the tumor are addicted to anaerobic glycolysis, others are addicted to pyruvate-dependent respiration [6–8].

The accumulation of a lot of data over the years on disorders of the regulatory pathways, especially those related to hypoxia and hypoxia inducible factor 1 alpha (HIF-1*α*), has led to a different explanation of the Warburg effect [11]. Thus, the accumulation of multiple dysregulations based on mutations is promoted and perceived as the root cause. Various oncogenes have been found to be upregulated and various tumor suppressor genes to be downregulated simultaneously in cancer cells of the same origin. This seems to have nothing to do with the metabolism itself, but only with the regulatory pathways of the gene expression. Moreover, it also indicates that at least several oncogenes and/or tumor suppressor genes must be affected simultaneously to effectively suppress cell proliferation.

When discussing the topic of cancer metabolism, the basic laws of thermodynamics should be considered due to the following important circumstance: Energy is what drives the biochemical processes in the respective direction, and the focus should be on the energy supply pathways! Proceeding from this circumstance, we try to answer the questions: Why is it assumed “a priori” that the genetic defects are the cause of cancer? Is not it possible to accept most of them as compensatory mechanisms by which cancer cells try to escape from some “energy trap” into which they have fallen? This “energy trap” forces them to grow and proliferate. Something that is energetically beneficial should guide their metabolism toward uncontrolled proliferation. Claiming “irreversibly impaired respiration”, Warburg only suggests that there must be a metabolic cause for the cancer, and it is rooted somewhere in the metabolism, associated with respiration.

In the last decade, cancer-type metabolism has been found to be typical for activated immune cells [12], as well as other proliferating noncancerous cells [13]. However, in these cells it should be reversible. It is still unknown what can be so fateful in the energy metabolism of cancer that pushes cells toward uncontrolled proliferation. If we try to understand this driving metabolic force that changes mitochondrial function and increases proliferation, then we can find the most important mutations, causing the irreversibility of cancer cell metabolism.

A decade ago, the main discussion in cancer cell metabolism was mainly focused on glycolysis, as well as genetic defects found in the Krebs cycle and mitochondria in general. Currently, there is a boom in studies, demonstrating that mFAO is vital for many types of cancer cells and they cannot exist without this metabolic pathway [14–17].

In this review, we propose the idea that many mysteries about the peculiarities of cancer cell metabolism, including respiration and even hypoxia, seem less inexplicable in the light of incomplete “combustion” of fatty acids in mitochondria.

Do glucose-dependent cancer cells use a huge amount of glucose just for glycolysis?

DeBerardinis et al. have calculated that glioblastoma cells convert more than 90% of the acquired glucose and ~60% of the acquired glutamine into lactate or alanine as by-products [18], but simultaneously they are characterized by a huge amount of NADPH produced from glucose and glutamine. Glucose energy is converted to NADPH energy in the pentose phosphate pathway (PPP). Glutaminolysis produces a large amount of NADPH by converting glutamate to malate, which in turn is converted to pyruvate by the malic enzyme.

In this review article, we consider the conversion of glutamate to malate as an important consequence from the overreduced state of mitochondrial matrix due to the high amount of NADH. Beta-oxidation should be involved in the production of this overreduced state. We consider the conversion of pyruvate to lactate as an important compensatory mechanism for diminishing the overreduced state of cancer cells and their survival.

On the other hand, NADPH energy could be used for many synthetic processes, and it is a major source of energy for fatty acid synthesis (FAS). It is well known that FAS is activated in many types of cancer cells [19–21] perhaps in all. Recently, it was demonstrated on glioblastoma cells that mFAO is expressed simultaneously with FAS [22, 23]. Is it possible to convert NADPH energy into ATP and how will the energy balance of the cell and its metabolism be affected if mFAO is expressed simultaneously with FAS, as found in glioblastoma model?

The analysis of the literature shows that the energy of NADPH can be converted into proliferative energy, as well as a certain amount of ATP. This process can be illustrated by the model of “artificial futile metabolic cycle” of “FAS+*β*-oxidation+citrate-malate shuttle” (see Section 10). The presumption is that cells do not necessarily have to use the same fatty acids for *β*-oxidation that they have synthesized. This model indicates that the energy stored by NADPH must be utilized primarily for lipid synthesis, to maintain the “*β*-oxidation shuttle” and mitochondrial function, not so much for ATP synthesis, but for synthesis of aspartate and citrate.

In our opinion, the “*β*-oxidation shuttle” can be expressed in the following circumstances: (i) impaired ETC and decreased utilization of NADH for ATP synthesis, (ii) decreased conversion of pyruvate to acetyl-CoA, and (iii) disturbed Krebs cycle in certain segments. In addition, *β*-oxidation can increase these three dysfunctions if it is overactivated beyond the needs of the cell for ATP.

Just to clarify that the article is focused only on mitochondrial *β*-oxidation.

## 2. Glucose Redirection to Pentose Phosphate Pathway and Lipid Synthesis

The pentose phosphate pathway (PPP) has been found to be overactive in cancer cells and multiple prooncogenic signaling pathways are involved in this process [24]. For example, normal tumor suppressor protein p53 has been found to inhibit glucose-6-phosphate dehydrogenase (G6PD), the first and rate-limiting enzyme of PPP [25]. Loss of p53 function due to mutations is a characteristic of most human cancers [26] and mutant p53 proteins do not inhibit G6PD [25].

What causes these cells to mutate so that PPP to be overactivated?

In general, high levels of PPP expression in cancer cells are related to the need of: (i) precursors for DNA synthesis and (ii) NADPH to quench high levels of reactive oxygen species (ROS) [24, 27].

Inflammation and oxidative stress are hallmarks of tumor tissues [28–30]. Oxidative stress can cause “glucose redirection to oxidative PPP in seconds” and this effect is achieved by ROS-dependent inhibition of glyceraldehyde 3-phosphate dehydrogenase (GAPDH) [31].

**Figure 1:**
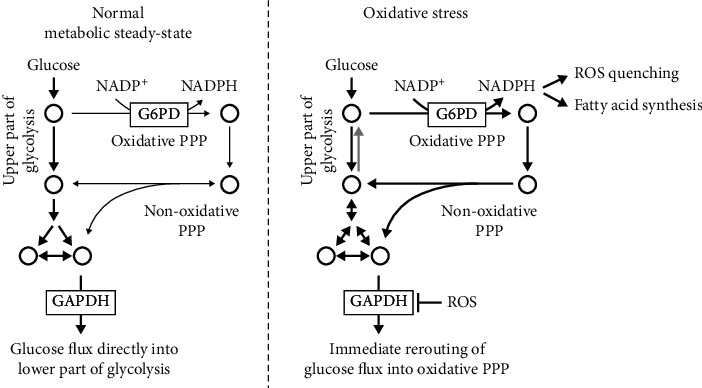
Metabolic flux in the pentose phosphate pathway (PPP) after oxidative stress and/or GAPDH inhibition in cells (adapted according to Kuehne et al. [31]). The metabolic rerouting in oxidative and nonoxidative PPP has important physiological roles in stabilization of the redox balance and ROS clearance. Acute activation of oxidative PPP is considered a first-line response to oxidative stress in cells. Oxidants induce rerouting of glucose flux into oxidative PPP within seconds. Initial rerouting is independent of GADPH inhibition. Multiple cycling of carbon compounds in the oxidative PPP potentially amplifies NADPH production. The metabolic PPP activation might be involved in resistance against ROS, particularly in cancer cells. Gray arrow indicates that in cancer cells this step is not reversible due to the loss of fructose-1,6-bisphosphate activity. The black blunt end indicates the inhibition of GAPDH. G6DH: glucose-6-phosphate dehydrogenase; GAPDH: glyceraldehyde-3-phosphate dehydrogenase; PPP: pentose phosphate pathway; ROS: reactive oxygen species.

Metabolic flux in PPP after oxidative stress and/or GAPDH inhibition has been also described [32]. Upon inhibition of GAPDH, PPP may continue to operate partially as a cycle due to reversibility of the nonoxidative part of PPP, as well as reversibility of the glucose-6-phosphate isomerase reaction (Figure 1). Out of context, it is interesting to note that many cancer cells have lost the gluconeogenesis enzyme, fructose-1,6-bisphosphatase [33]. This enzyme appears to be crucial for the glycolytic behavior of cancer cells [33].

GAPDH inhibitors are promising candidates for the treatment of highly glycolytic cancers [32, 34–36]. However, resistance can develop independently of drug metabolism or intracellular transport. Liberti et al. have demonstrated that BT-549 breast cancer cells become resistant to GAPDH inhibitor, koningic acid, after prolonged treatment [37]. Koningic acid-resistant cancer cells lose their Warburg effect, measured as lactic acidosis. However, these cells remain dependent on glucose and become dependent on lipid metabolism.

The fact that cancer cells may lose their dependence on glycolysis, but remain dependent on glucose, suggests that lactate production is not as important to them as NADPH production.

It should be noted that cancer cells are also characterized by altered lipid metabolism and overactivation of FAS is a distinctive feature [15, 19, 21]. FAS depends on the energy of reducing equivalents such as NADPH synthesized mainly in PPP.

Above are examples of how the glucose energy can be used to produce something other than pyruvate, such as NADPH. In this case, the energy stored in NADPH can be used directly to produce fatty acids. Can this energy be converted into ATP?

## 3. The Importance of FAS and mFAO for Cancer Cells

Enhanced synthesis or uptake of lipids contributes to rapid cancer cell growth and tumor progression [38, 39].

Cancer cells are characterized by high cytoplasmic glucose levels, mainly due to overexpression of glucose transporters [40]. In normal cells, regulatory mechanisms activate FAS in the case of high cytoplasmic glucose levels. If cancer cells had this normal metabolism, they would simply convert glucose into fatty acids (as in the case of normal hepatocytes) and the tumor tissue would not accumulate lactate but triglycerides. In contrast, in glioma cells, it has been shown that 90% of glucose is converted to lactate as an end-product [18], although these cancer cells are characterized by overactivated FAS [20, 41].

Fatty acids are synthesized from cytoplasmic acetyl-CoA and NADPH. Where does acetyl-CoA come from for overactivated FAS in cancer, particularly in glioma?

In fact, mFAO is the best source for production of acetyl-CoA. FAS is elevated in glioma cells, but the same is true for mFAO [20, 22, 23]. In gliomas, FAS and mFAO inhibitors suppress the proliferation of cancer cells, suggesting the existence of the “futile cycle” mentioned in the Introduction [41]. Moreover, some cancer cells prefer to be located close to adipocytes—the main store of fats [42]. In 2011, Nieman et al. found that coculturing ovarian cancer cells with human-derived adipocytes resulted in increased *β*-oxidation by activating CPT1 and acyl-CoA oxidase 1 [43]. This made *β*-oxidation an interesting new target for the treatment of ovarian cancer. At the same time, the inhibitors of fatty acid synthase (encoded by the FASN gene) cause growth arrest and apoptosis in the same type of cells [44]. FASN is the rate-limiting enzyme of FAS, which is overexpressed in ovarian cancer.

Numerous studies discussed below have demonstrated the significant role of irregularly activated mFAO in the metabolism and survival of cancer cells. One of the first studies on this topic was published in 2009 [45], and shortly thereafter, other studies reported the same [46, 47]. Some of these studies have been analyzed and highlighted in a review article on the role of mFAO in cancer, suggesting the possible coexistence of FAS and mFAO [48].

In 2013/2014, it was reported that acidosis leads to a decrease in the glucose consumption by cancer cells and an increase in the anaplerotic pathways of the Krebs cycle [49, 50]. In this case, activation of mFAO could be suggested due to decreased glucose consumption (see Section 5). A decrease of glucose uptake by an increase of mFAO is a well-known regulation, associated with the “Randle cycle”.

In 2014, Schlaepfer et al. demonstrated that the CPT1 inhibitor, etomoxir, suppressed proliferation of prostate cancer cells (LNCaP) and tumor growth in nude mice [51]. The same effect was observed in CPT1A-knockdown LNCaP cells, which was accompanied by reduced oxidation of palmitate.

In 2016, Corbet et al. discovered how mFAO could be irregularly activated in acidosis-adapted cancer cells. The authors found that deacetylation of histones in cancer cells (adapted to acidic pH) leads to a decreased expression of acetyl-CoA carboxylase 2 (ACC2) [52], whose activity is the main regulatory factor for mFAO inhibition. They also observed а significant nonenzymatic acetylation of mitochondrial proteins, caused by elevated levels of acetyl-CoA. In turn, this leads to acetylation and inactivation of complex I in the mitochondrial ETC. This relationship between acidification and *β*-oxidation was called “Corbet-Feron effect” [41], which hints at its universality.

Recently, Luis et al. reported that under certain conditions, such as obesity-mimicking status, cancer cells can spend lipids and amino acids to synthesize glucose from lactate via gluconeogenesis [53]. The authors have called this phenomenon “Warburg effect inversion”. The authors cultured breast cancer cells (MCF-7) in three different media—high-glucose concentration, low-glucose concentration, and high concentration of fatty acids released by adipocytes. The medium containing adipocytes significantly increases the viability and proliferation of MCF-7 cells, as well as their migration rate and aggression. Cancer cells in a high-fat environment excrete significant amounts of glucose, pyruvate, and acetate. Here, it is important to note that a distinctive feature of activated mFAO (in this case) is the increased consumption of lactate and glutamine.

Another mechanism for overactivation of mFAO in cancer cells is through modulation of ACC2 activity by hydroxylation [54]. Prolyl hydroxylase 3 has been found to hydroxylate and activate ACC2, whose malonyl-CoA product inhibits mFAO. Prolyl hydroxylase 3 is an oxygen sensor, known as а deactivator of HIF-1*α*. This enzyme is essential for regulation of hypoxia. In the presence of oxygen, prolyl hydroxylate 3 hydroxylates HIF-1*α* and deactivates it by directing it to ubiquitination. The finding that prolyl hydroxylase 3 also hydroxylates and activates the metabolic enzyme ACC2 means that at normal oxygen levels mFAO should rather be inhibited. However, in acute myeloid leukemia (AML) cells, the level of prolyl hydroxylase 3 has been found to be low and thus potentiates their dependence on mFAO in normoxia [54]. Other mechanisms for downregulating ACC2 have also been described [55].

The studies mentioned above demonstrate that mFAO can be overactivated by suppressing ACC2 under acidic conditions, reduced prolyl hydroxylase 3 activity in hypoxia, or by increased availability of fatty acids, and these are very important circumstances in cancer metabolism. This mechanism appears to be universal, given that the acidic environment of cancer cells and hypoxia are common features of tumors and can activate mFAO. The same goes for the link between cancer and diabetes [56, 57].

Increased expression of the MYC oncogene, which is a hallmark of many human cancers, is also associated with their metabolism [58, 59]. Impairment of respiratory chain by MYCN inhibitors results in the accumulation of cytoplasmic lipid droplets [60]. Apparently, impaired respiration can stop the consumption of citrate in the Krebs cycle, and this leads to the accumulation of cytoplasmic citrate and subsequent activation of FAS. However, MYC-overexpressing tumors, such as triple negative breast tumors, have been found to be addicted to fatty acid oxidation, and pharmacological inhibition of fatty acid oxidation blocks their growth [61].

At present it is difficult to predict how many cancers are dependent on mFAO. Most likely, these are cancers that have been shown to be oxidative. Amoedo et al. identified two subgroups of lung carcinomas—high and low OXPHOS-expressing tumors [62]. The high OXPHOS-expressing tumors poorly incorporated fluorodeoxyglucose (18F) and had increased expression of the mitochondrial trifunctional fatty acid oxidation enzyme (TFP; particularly TFP subunit alpha) compared to the paired adjacent tissue. Genetic and pharmacological inhibition of TFP subunit alpha affects tumor growth in vivo. Trimetazidine, an approved drug inhibitor of TFP used in cardiology, disrupts the interaction between the TFP and complex I of the ETC, leading to a cellular redox and energy crisis [62].

Mitochondrial FAO is considered an important factor in the growth of cancer cells, as well as an important target in the development of new therapeutics for various cancers such as pancreatic [63], prostate [64], leukemia [47, 65], lymphoma [66], and ovarian [43].

The transcriptional coactivator yes-associated protein (YAP) is the other factor that upregulates genes from the mFAO signaling pathway. Tumor lymph node metastases were found to be associated with YAP activation, respectively mFAO activation [67]. Genetic ablation of YAP or inhibition of mFAO suppresses lymph node metastases in mice. The expression of the main protein of mFAO, the TFP subunit alpha encoded by HADHA gene, has been shown to be a useful marker for predicting resistance to platinum-based chemotherapy in patients with lung cancer [68]. Recent articles have focused on mFAO in cancer cells and its association with metastasis [14, 17, 69, 70].

Studies on mFAO do not cover yet many different types of cancer, but publications on this phenomenon have increased enormously over the last decade and mFAO inhibitors have been successful in treating cancer cells [69, 71–74].

The growing evidence of overactivated mFAO in cancer cells in addition to abnormally activated metabolic pathways such as FAS and PPP suggest for the existence of a metabolic cycle, in which FAS coexists with mFAO. This cycle is prohibited in normal cellular metabolism under normal conditions.

## 4. mFAO in Normal Cells Is Regulated to Work Only in Energy Deficiency

The usual thought is that fats are a reserve fuel, and they are used only in case of energy deficiency. However, there is no answer to the question: “Why is that? Is this just an idea coming from empirical observations or there is a deep biochemical reason?”

**Figure 2:**
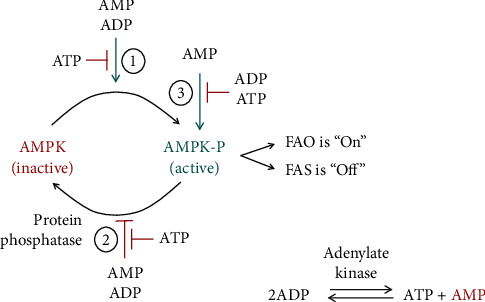
Regulation of AMPK activity—role of ATP/ADP ratio and AMP level (adapted according to Hardie and Alessi [75]). The red blunt ends indicate the inhibition of a corresponding process. AMPK: 5′AMP-activated protein kinase (dephosphorylated state—inactive); AMPK-P: 5′AMP-activated protein kinase (phosphorylated state—active); FAO: fatty acid oxidation; FAS: fatty acid synthesis.

In general, at a normal glucose concentration, the most important energy indicator in cells, the ATP/ADP ratio is high. AMP-activated protein kinase (AMPK) is the main sensor of this ratio, and this enzyme is not active at normal or high ATP/ADP ratio (Figure 2) [75].

**Figure 3:**
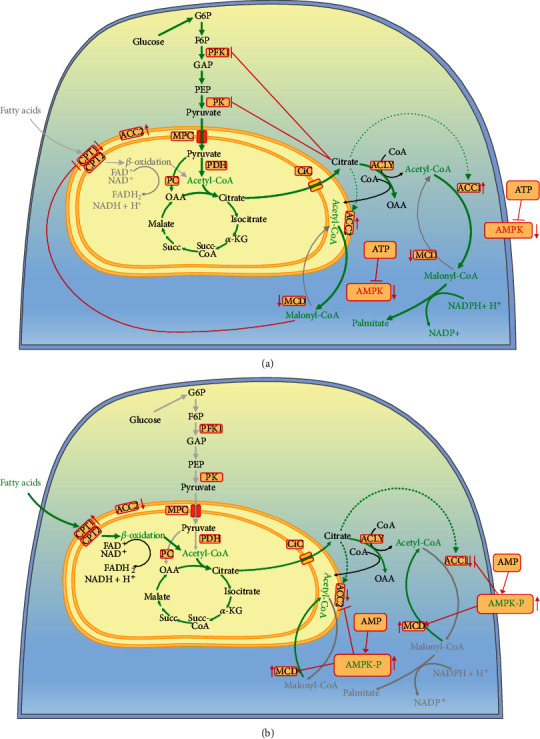
(a) Regulation of FAS and mFAO by AMPK at normal or high ATP/ADP ratio. In this case, AMPK is in inactive state, ACC1 and ACC2 are active, and the accumulated malonyl-CoA inhibits the *β*-oxidation of fatty acids in the mitochondria by suppressing CPT1 and CPT2 activity. (b) Regulation of FAS and mFAO by AMPK at low ATP/ADP ratio. In this case, AMPK is in an active state (AMPK-P), ACC1 and ACC2 are deactivated, malonyl-CoA is removed by activated MCD, which eliminates the inhibition of CPT1 and CPT2 and activates the *β*-oxidation of fatty acids in the mitochondria. The green arrows indicated the expressed pathway. The gray arrows indicate the suppressed pathway. The red blunt ends indicate the inhibition of a particular enzyme. The red arrows indicated the activation of a particular enzyme. ACC1 and ACC2: acetyl-CoA carboxylases 1 and 2; ACLY: ATP citrate lyase; AMPK: 5′AMP-activated protein kinase (dephosphorylated state); CIC: mitochondrial citrate carrier; CTP1 and CTP2: carnitine palmitoyl transferases 1 and 2; F6P: fructose-6-phosphate/fructose-1,6-bisphosphate; G6P: glucose-6-phosphate; GAP: glyceraldehyde-3-phosphate; a-KG: a-ketoglutarate; MCD: malonyl-CoA decarboxylase; MPC: mitochondrial pyruvate carrier; OAA: oxaloacetate; PDH: pyruvate dehydrogenase; PC: pyruvate carboxylase; PFK1: phosphofructokinase-1; PEP: phosphoenolpyruvate; PK: pyruvate kinase; Succ: succinate.

When AMPK is deactivated, FAS is activated and mFAO is deactivated, which is modulated by the activity of acetyl-CoA carboxylases ACC1 and ACC2 (Figure 3(a)). ACC1 is located in the cytoplasm and is responsible for the synthesis of malonyl-CoA, which is mainly involved in the synthesis of palmitate. ACC2 is located in the intermembrane mitochondrial space and is involved in the synthesis of malonyl-CoA, which inhibits the key regulatory enzyme of mFAO, CPT1, and thus inhibits the *β*-oxidation of long-chain fatty acids in mitochondria [76]. The presence of ACC2 in the intramembrane space of mitochondria is important for the inhibition of CPT1 by malonyl-CoA in the same proximity [77].

At normal or high ATP/ADP ratio, AMPK is not activated and in turn, ACC1 and ACC2 are in their active dephosphorylated and polymer forms [78]. The metabolite citrate, which is required for FAS, appears in the cytoplasm only if the glucose in the cell exceeds the energy needs, but cannot appear as a result of mFAO, because the AMPK-ACC2 mechanism turns “OFF” mFAO at normal ATP level. Citrate is an inhibitor of two glycolytic enzymes (PFK1 and PK), which is a feedback mechanism to stop the overcharging of cells with energy and precursors for synthetic processes. Citrate will inhibit glycolysis only if its concentration exceeds the capacity of the enzyme citrate lyase (ACLY). ACLY is primarily regulated at the genetic level and its activity does not appear to depend on a metabolite other than citrate [79]. At the same time, citrate is an activator of ACC1 and ACC2. This may lead to depletion of citrate in the cytoplasm by removing the product from the citrate lyase reaction, acetyl-CoA (Figure 3(a)). If the citrate is depleted by conversion to acetyl-CoA, then all the excess glucose will be directed to its conversion into fatty acids. The first step in FAS is the conversion of acetyl-CoA to malonyl-CoA. Malonyl-CoA should be considered primarily as a trigger of FAS, a precursor to FAS, and an inhibitor of mFAO. Therefore, it is normal for malonyl-CoA to be in relatively high concentrations in the cells when glucose is in excess. In a fed state, the normal concentration of malonyl-CoA in cells is thought to be about 200 *μ*moles [80]. This is also the concentration at which malonyl-CoA inhibits CPT1 [80].

At extra glucose in normal cells, all excess cytoplasmic citrate will be converted to malonyl-CoA or accumulate to the threshold of inhibition of its source—glycolysis (Figure 3(a)). The type of cells determines which of these two events will occur: (i) in cells strongly expressing enzymes for lipid synthesis (for example, fatty acid synthesis), lipid synthesis will dominate and (ii) in cells weakly expressing enzymes for lipid synthesis, cytoplasmic citrate accumulation and glycolysis inhibition will dominate. The existence of the enzyme malonyl-CoA decarboxylase (MCD) seems useless in the case of active glucose degradation (Figure 3(a)) but makes sense in the case of a rapid change in the direction of glucose deficiency. When glucose is depleted and the main energy indicator, the ATP/ADP ratio decreases, the ADP concentration increases, and AMP appears because of the function of the enzyme adenylate kinase (Figure 2). AMPK is activated and ACC1 and ACC2 are inactivated (Figure 3(b)). In this case, malonyl-CoA should be removed by the activated MCD to rapidly eliminate the inhibition of CPT1 and to activate mFAO. This activates mitochondrial *β*-oxidation and stops the loss of energy from fatty acid resynthesis (Figure 3(b)). Subsequent recovery of the normal ATP/ADP ratio should return the cell to its normal state by deactivating AMPK, activating ACC1 and ACC2, and deactivating MCD. This restores the normal dependence of the cell on glucose, if available.

It is important to note that citrate is unlikely to accumulate in the cytoplasm under activated *β*-oxidation and low ATP levels, respectively in the presence of AMP. Under these conditions, most acetyl-CoA should be consumed in the Krebs cycle. However, after the appearance of sufficient amount of ATP in the cellular environment, mFAO should be deactivated by the AMPK-ACC2 mechanism.

What happens if this mechanism is dysregulated and does not turn “OFF” the mFAO, when glucose is available again?

## 5. Overactivated Mitochondrial *β*-Oxidation Inhibits Glycolysis and May Suppress the Krebs Cycle

**Figure 4:**
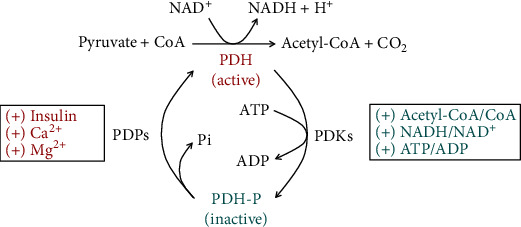
Regulation of pyruvate dehydrogenase (PDH) activity by phosphatases and kinases (adapted according to Bhandary and Aguan [85]). The enzyme activity of pyruvate dehydrogenase phosphatases (PDPs) depends on the intramitochondrial concentration of Mg^2+^, Ca^2+^, and insulin. The enzyme activity of pyruvate dehydrogenase kinases (PDKs) depends on the ratios Acetyl-CoA/CoA, NADH/NAD^+^, and ATP/ADP.

Cancer is associated with many disorders such as acidosis, hypoxia, and diabetes, but obesity-related diabetes is the most studied phenomenon over time and closest to fatty acid metabolism. In diabetes, the antagonism between fat and sugar combustion is the most discussed metabolic disorder, which has attracted Randle's attention [81, 82]. The basic concept in Randle's publications is “inhibition of glycolysis by activating mitochondrial *β*-oxidation”. If hormonal regulations in the organism are excluded, the simple activation of *β*-oxidation in mitochondria at high levels of fatty acids leads to the activation of several mechanisms that inhibit glycolysis and utilization of pyruvate in the Krebs cycle [82]. Some of these mechanisms are (i) intramitochondrial inhibition of PDH by accumulation of acetyl-CoA and NADH, (ii) activation of pyruvate carboxylase (PC) by increased acetyl CoA, and (iii) increased NADH/NAD^+^ ratio [83]. PDH activity was also found to be regulated through reversible phosphorylation by PDH kinases (PDKs) and PDH phosphatases (PDPs) [84]. PDK is activated by the main products of *β*-oxidation—acetyl-CoA and NADH (Figure 4). Activated PDK phosphorylates and inactivates PDH.

**Figure 5:**
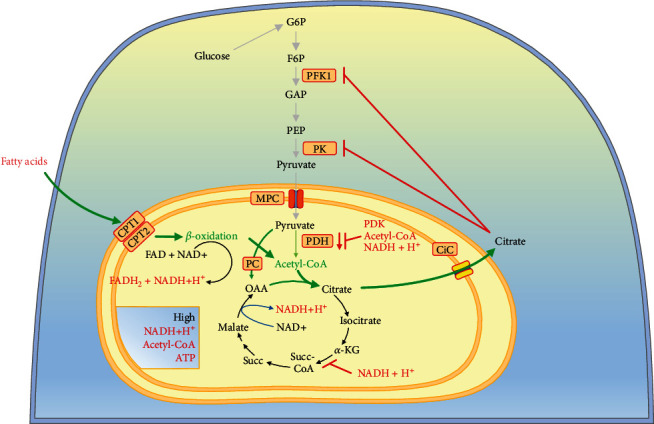
Antagonism between mFAO and glycolysis and accumulation of acetyl-CoA from fatty acid oxidation (adapted according to De Oliveira and Liesa [70]). Overactivated mitochondrial *β*-oxidation inhibits glycolysis and may also suppress the Krebs cycle. The main consequences are increased mitochondrial ratios of NADH/NAD^+^, acetyl-CoA/CoA, ATP/ADP, as well as citrate accumulation in the mitochondria. The green arrows indicated the expressed pathway. The gray arrows indicate the suppressed pathway. The red blunt ends indicate the inhibition of a particular enzyme. ACC1 and ACC2: acetyl-CoA carboxylases 1 and 2; ACLY: ATP citrate lyase; AMPK: 5′AMP-activated protein kinase (dephosphorylated state); CIC: mitochondrial citrate carrier; CTP1 and CTP2: carnitine palmitoyl transferases 1 and 2; F6P: fructose-6-phosphate/fructose-1,6-bisphosphate; G6P: glucose-6-phosphate; GAP: glyceraldehyde-3-phosphate; a-KG: a-ketoglutarate; MCD: malonyl-CoA decarboxylase; MPC: mitochondrial pyruvate carrier; OAA: oxaloacetate; PDH: pyruvate dehydrogenase; PC: pyruvate carboxylase; PFK1: phosphofructokinase-1; PEP: phosphoenolpyruvate; PK: pyruvate kinase; Succ: succinate.

The accumulation of citrate in the mitochondria is a major consequence of the accumulation of acetyl-CoA, but also depends on the presence of oxaloacetate [86]. The presence and activation of mitochondrial pyruvate carboxylase increases citrate production through the Krebs cycle anaplerosis. Export of citrate from mitochondria and its accumulation in the cytoplasm also lead to inhibition of several glycolytic enzymes [84] (Figure 5).

The main aspect of these pioneering studies is the increased mitochondrial ratios of NADH/NAD^+^, acetyl-CoA/CoA and ATP/ADP, and the accumulation of citrate in the mitochondria [70] (Figure 5). Currently, the study of antagonism between mFAO and glycolysis and accumulation of acetyl-CoA from fatty acid oxidation seems to have been forgotten. Even less attention is paid to the increased NADH/NAD^+^ ratio [83]. The increase in NADH has not been explained and has also been forgotten. The Krebs cycle could be inhibited by elevated NADH concentrations. High amount of NADH inhibits the PDH and Krebs cycle dehydrogenases and decreases combustion of pyruvate and glucose [87]. This is normal regulation when the amount of ATP is at the upper threshold in the cell, which decreases glucose combustion. Does this regulation also work for mFAO to decrease fatty acid combustion? The answer seems to be positive, given that the Krebs cycle can be inhibited by high concentrations of NADH.

However, mFAO consists of two parts—*β*-oxidation and the Krebs cycle. ATP does not apply the same force to *β*-oxidation, which precedes the Krebs cycle. We are accustomed to accept that at rest, the energy metabolism is self-regulating on the principle of feedback and the NADH/NAD^+^ ratio in mitochondria may vary depending on the utilization of ATP. Sahlin and Katz reported that mitochondrial NADH in skeletal muscles at rest is between 36% and 60%, while in the heart muscles it is between 4.2% and 13% [88]. So, that must be the difference in the redox state of the mitochondrial matrix between highly active tissues and tissues in rest. In rest, ATP has a feedback effect on all metabolic processes associated with its production. This also applies to the Krebs cycle.

In the classic studies of Randle and other authors, as in all cases of mFAO in cancer cells that we have already discussed, the same phenomenon is observed: overactivated mFAO, exceeding the energy needs of the cell, as the AMPK-ACC2 mechanism is avoided or disrupted. In diabetes, this regulatory mechanism is avoided by high concentrations of fatty acids, which is common situation in this disease. In cancer, the AMPK-ACC2 mechanism is disrupted due to impaired regulation of AMPK signaling or altered ACC2 activity, as discussed above. The limited data on ATP concentrations in cancer cells suggest that these cells are not in energy deficiency, and they have more ready-to-use ATP levels than their normal counterparts [89].

## 6. Other ATP-Dependent Feedback Mechanisms to Stop Overactivated *β*-Oxidation

The data presented in the previous section suggest a very important conclusion: The exclusion of mFAO when the intracellular ATP/ADP ratio normalizes is entrusted to the AMPK-ACC2 mechanism. This is a completely different way of stopping the mitochondrial combustion of fatty acids, compared to the case where pyruvate is the main fuel.

**Figure 6:**
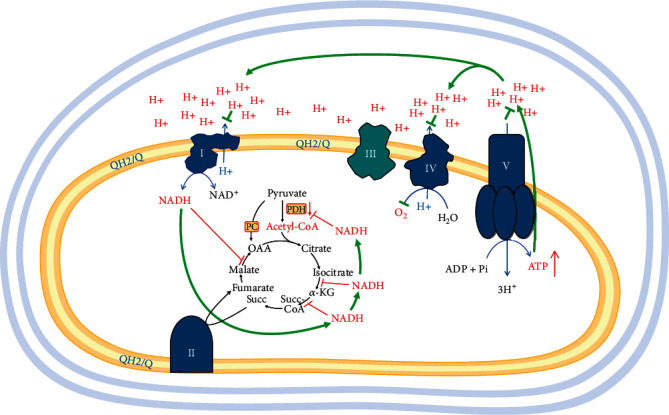
ATP-dependent mechanism for stopping combustion of pyruvate and glucose in mitochondria, mediated by high amounts of NADH. The green arrows indicated the accumulated metabolites. The red blunt ends indicate the inhibition of a particular enzyme. a-KG: a-ketoglutarate; OAA: oxaloacetate; Q: coenzyme Q10 (oxidized form); QH2: coenzyme Q10 (reduced form); PDH: pyruvate dehydrogenase; PC: pyruvate carboxylase; Succ: succinate; Succ-CoA: succinyl-CoA; VLCDH: very long-chain acyl-CoA dehydrogenase.

When pyruvate is combusted to CO_2_ and H_2_O in the Krebs cycle, there are no substantial end products of the overall reaction that can inhibit this process other than ATP. This is a prerequisite for mitochondrial ATP to directly inhibit pyruvate combustion by inhibiting respiration. It should be noted that in this case, the cell relies entirely on mitochondrial ATP as a feedback mechanism, and this is the only factor that controls the Krebs cycle and the assimilation of pyruvate (Figure 6).

It is worth thinking about: “Why, under normal conditions, the AMPK-ACC2 mechanism starts mFAO only in the case of ATP deficiency, but turns it “OFF” as soon as the ATP level is restored?” (Figure 3(b)).

The feedback mechanism for regulating glucose metabolism by ATP involves inhibition of the Krebs cycle by NADH, which is not consumed by complex I. In this article, we propose the idea that elevated levels of ATP and NADH do not have this power to inhibit mFAO itself and specifically the part of mFAO preceding the Krebs cycle—*β*-oxidation.

**Figure 7:**
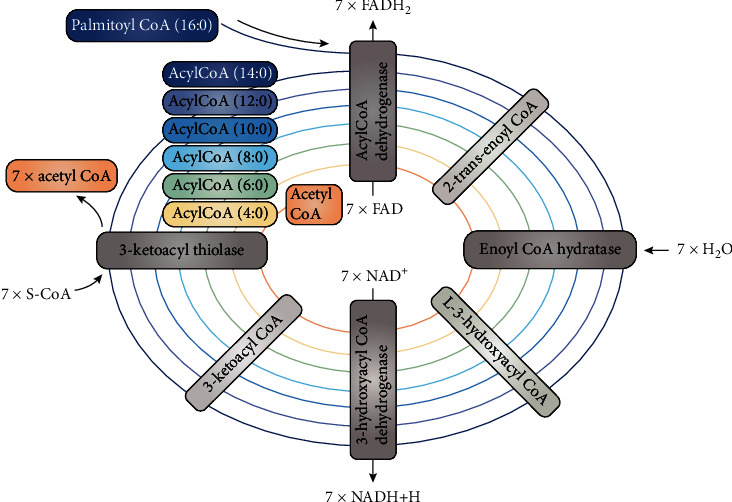
Representation of the *β*-oxidation of palmitic acid in the mitochondria (according to Carracedo et al. [48], with permission of the Nature Publishimg Group). Acyl-CoAs enter the fatty acid oxidation pathway in which they are dehydrogenated, hydrated, and decarboxylated cyclically. This results in the progressive shortening of the fatty acid. The produced NADH and FADH_2_ can be used for ATP production in the electron transport chain, and acetyl-CoA can enter the Krebs cycle. S-CoA: free coenzyme A.

Mitochondrial *β*-oxidation is carried out by 4 types of enzymes that catalyze the 4 types of reactions from this spiral process for digestion of fatty acids containing n-number of carbon atoms to n/2-number of acetyl-CoA [90] (Figure 7):

(1) Different acyl-CoA dehydrogenases (ACADs) with different affinities for fatty acids with different chain lengths. ACADs are FAD dependent and tightly connected with the internal mitochondrial membrane. They produce trans-2-enoyl-CoA with different chain lengths. ACADs transfer electrons from fatty acids to a short electron transfer pathway. This pathway consists of “electron transfer flavoprotein” (ETF) and “electron transfer flavoprotein-ubiquinone oxidoreductase” (ETF-QO) and transfers electrons from ACADs to coenzyme Q [91]
(2) Four groups of enzymes, forming mitochondrial trifunctional protein [92]
  - Several enoyl-CoA hydratases (EHs) with different affinities for trans-2-enoyl-CoA with different length. Thus, EHs produce L-3-hydroxyacyl-CoA with different chain lengths
  - Several 3-hydroxyacyl-CoA dehydrogenases (3HADs) that convert 3-hydroxyacyl-CoA to 3-ketoacyl-CoA and produce NADH from NAD^+^
  - At least three 3-ketothiolases (KATs) that convert 3-ketoacyl-CoA into acetyl-CoA and two carbons shorter acyl-CoA

The complete oxidation of one molecule of saturated acyl-CoA, such as palmitoyl-CoA, must go through a series of chain length-specific ACADs, EHs, 3HADs, and KATs to catalyze the cyclic release of acetyl-CoA units. Only two of the four reactions are oxidative. The reactions catalyzed by FAD-dependent ACADs cannot be directly inhibited by ATP, high transmembrane potential, or high levels of FAD. No ATP binding sites have been reported for these enzymes, they are not proton pumps and are independent of the transmembrane potential, and FAD is a prosthetic group in these enzymes.

The 3HAD enzymes that catalyze the second step of *β*-oxidation are NAD^+^ dependent, and because NAD^+^ and NADH are cofactors, their concentrations may affect enzyme activity. In 1998, Eaton et al. investigated the sensitivity of isolated mitochondrial TFP activity to two different concentrations of end-products of *β*-oxidation, NADH and acetyl-CoA [93]. The authors found that the TFP was relatively insensitive to NAD^+^/NADH ratio. Inhibition of its enzyme activity begins at a NAD^+^/NADH ratio of less than 1 or 2.5 (when NADH increases above 50-60%). To recall the study of Sahlin and Katz, which shows that mitochondrial NADH in skeletal muscle at rest does not exceed 60%, and in active heart muscle it does not exceed 13% [88]. Therefore, it can be concluded that the TFP cannot be inhibited at physiological concentrations of NADH. In comparison, *α*-ketoglutarate dehydrogenase (*α*-KGDH), a key enzyme in the Krebs cycle, is almost completely inhibited by 50% NADH and its activity decreases more than 2 times at 25% NADH [94, 95]. Inhibition of NAD-dependent isocitrate dehydrogenase (NAD-IDH) is even more pronounced [96]. IC_50_ values for different isoforms of NAD-IDH vary between 7.8% and 9.4% of NADH versus NAD^+^ and reach almost complete inhibition at 35-40% of NADH [96].

**Figure 8:**
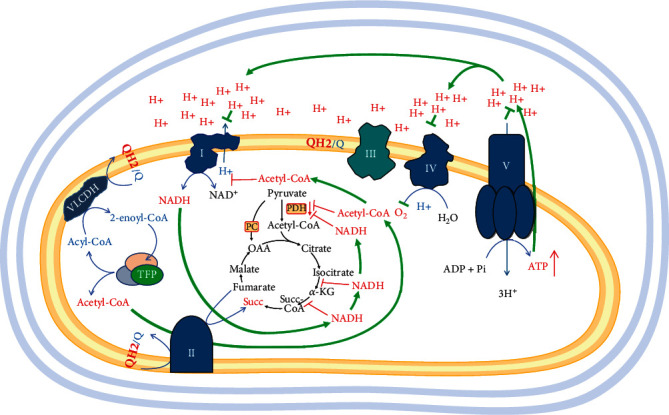
Possible consequences of the overactivated *β*-oxidation in mitochondria: high Q10H_2_/Q10 ratio and accumulation of acetyl-CoA and succinate. This could lead to high levels of NADH and acetyl-CoA that inhibit the Krebs cycle, PDH, and complex I of the mitochondrial ETC. The green arrows indicated the accumulated metabolites. The red blunt ends indicate the inhibition of a particular enzyme. a-KG: a-ketoglutarate; OAA: oxaloacetate; Q: coenzyme Q10 (oxidized form); QH_2_: coenzyme Q10 (reduced form); PDH: pyruvate dehydrogenase; PC: pyruvate carboxylase; Succ: succinate; Succ-CoA: succinyl-CoA; TFP: trifunctional protein; VLCDH: very long-chain acyl-CoA dehydrogenase.

If the concept that ATP and NADH do not have much power to inhibit the spiral process of mitochondrial *β*-oxidation is true, the result from overactivated process will be an accumulation of coenzyme Q10 in reduced form (Q10H_2_), succinate, NADH, and acetyl-CoA, as described in Randle's and more recent publications [70, 84, 97]. Therefore, if there is a feedback mechanism to stop mitochondrial *β*-oxidation, which is an alternative to the AMPK-ACC2 mechanism when it is violated, we should look for it in relation to these four products (Figure 8).

The next chapters highlight and analyze many studies, demonstrating that the accumulations of these four metabolites are common characteristics of cancer cells and that mitochondrial *β*-oxidation is most likely involved in their production.

### 6.1. Q10H_2_/Q10 Ratio and Succinate Accumulation

The assumption that *β*-oxidation may lead to a higher Q10H_2_/Q10 ratio seems intuitive, but Q-pool overcharging occurs in hypoxia and/or functional deficiency of ETC complexes. Hypoxia and/or ETC-complex deficiency prevent the utilization of electrons, while *β*-oxidation supplies ETC with electrons. If *β*-oxidation cannot be inhibited by higher NADH concentrations in the mitochondria, it should increase the Q10H_2_/Q10 ratio when electrons are not consumed.

Recently, Guarás et al. found that the shift from glucose to fatty acid metabolism increases electron flux through FAD-dependent enzymes, which saturates the Q-pool and leads to reverse electron transport (RET) through complex I [97]. In addition, this is accompanied by downregulation of complex I. It is not clear whether this downregulation occurs through the direct destruction of complex I by increased ROS production or by ROS-mediated regulatory pathways. Their results support the idea that coenzyme Q10 (Q10) redox state acts as a metabolic sensor that fine-tunes the mitochondrial ETC configuration to adjust the electron flux to the FADH_2_/NADH ratio. The calculated FADH_2_/NADH ratio is quite different when fatty acids are the main fuel of ETC instead of glucose [98]. In the case of glucose as a fuel and active malate-aspartate shuttle, this ratio is 0.2 (one FADH_2_ molecule per five NADH molecules). In the case of saturated long-chain fatty acids as a fuel, this ratio is approaching 0.5 (one FADH_2_ molecules per two NADH molecules) [98].

Can the Q10H_2_/Q10 ratio have a direct inhibitory effect on mitochondrial *β*-oxidation?

Very long-chain acyl-CoA dehydrogenase (VLCAD) is the first enzyme involved in the *β*-oxidation pathway. It is also the first part of the short electron transfer chain, transferring electrons from the substrate acyl-CoA to coenzyme Q10 via the ETF and ETF-QO proteins [91]. A feedback redox effect is possible through a protein-protein interaction between these components of the redox chain. VLCAD also appears to have other regulatory functions. For example, the activity of VLCAD has been found to be modulated by myeloid leukemia cell differentiation protein (MCL-1)—a member of B cell lymphoma 2 family antiapoptotic proteins that are overexpressed in cancer cells [99]. MCL-1 causes increased metabolic flux through mFAO in response to nutrient deprivation, and this supports the assumption of enhanced mFAO in cancer cells with higher expression of MCL-1.

Currently, there is only indirect evidence that feedback can exist. In 2005, Mason et al. discovered that the first stage of *β*-oxidation can be inhibited directly due to ischemia [100]. They found that VLCAD activity decreased by 34% during 30 min of ischemia. The loss of activity appears to be specific for VLCAD, as the activity of medium-chain acyl-CoA dehydrogenase (MCAD) remains constant. The loss of VLCAD activity during ischemia is not due to loss of protein content. The activity is restored in the presence of detergent Triton X-100. This suggests that changes in the interaction between the VLCAD and the inner mitochondrial membrane are responsible for the ischemia-induced loss of its enzyme activity. However, MCAD activity was not affected by hypoxia. Based on our knowledge, there is no other evidence that the redox state of mitochondrial ETC could directly affect the activity of mFAO enzymes, and we cannot currently confirm that inhibition of VLCAD could be mediated by the Q10H_2_/Q10 ratio. Even if such a mechanism exists, we cannot answer the question: “When this mechanism is activated - before or after the accumulation of large amounts of metabolites (such as succinate, NADH, and acetyl-CoA) in the matrix?” The answer to this question is important because the excessive accumulation of acetyl-CoA and NADH can lead to serious metabolic rearrangements of mitochondria.

The increased Q10H_2_/Q10 ratio at overactivated *β*-oxidation could be easily combined with hypoxia, a common event in cancer environment. Hypoxia and *β*-oxidation can insert complementary effects on the Q10H_2_/Q10 ratio and the Krebs cycle. Mitochondrial ETC and the Krebs cycle linked by the enzyme succinate-coenzyme Q reductase (SQR) or also called succinate dehydrogenase (SDH) for short (Figure 8), and the Q-pool is the connecting link. SDH catalyzes the conversion of succinate to fumarate. The succinate dehydrogenase reaction is thermodynamically reversible [101]. Accumulation of succinate in cancer cells is a common event and is often explained by inhibitions or mutations of SDH [102]. However, in cancer cells with functional SDH, the highly reduced Q-pool should have the same impact.

In plant mitochondria, it was found that SDH activity is directly related to the Q-pool redox balance [103]. If the same is valid for mammalian cells, then the notion that SDH will be inhibited in a highly reduced Q-state is a logical assumption.

In 2009, Tomitsuka et al. discovered fumarate reductase activity of complex II in five different types of cancer cells. The authors reported that this activity was low but increased significantly when cells were cultured under hypoxic and glucose deprived conditions [104]. They explained the backward activity of enzyme by different phosphorylation states of SDH. However, they did not investigate the possible increase of the Q10H_2_/Q10 ratio or the activation of mFAO in case of glucose deprivation. Protein modification may increase or decrease enzyme activity, but phosphorylation does not have sufficient driving force to reverse the enzyme reaction. Currently, it is accepted that in acute hypoxia, the SDH reaction is reversed, and the driving force of this reversal is the accumulated NADH, which cannot be consumed by the ETC. This is accompanied by an increase in the Q10H_2_/Q10 ratio because Q10 is a mediator in the transfer of electrons from NADH to succinate in hypoxia. In 2012, the same authors demonstrated that glucose deprivation without hypoxia also induces reversal of SDH-reaction [105]. It appears that *β*-oxidation, which is assumed to be activated in glucose deprivation, has almost the same power to reduce the Q-pool as accumulated NADH in hypoxia.

The reversibility of reactions of the Krebs cycle is a revolutionary concept that is beginning to be discussed by scientists [101]. In the case of activated *β*-oxidation, it is quite possible for the Krebs cycle to be inhibited at the succinate segment by increasing the thermodynamic force and significantly decreasing the SDH activity, which will be accompanied by the accumulation of succinate.

### 6.2. Succinate Accumulation as а Common Feature of Cancer Cells and Overactivated mFAO

The excretion of succinate from cancer cells is considered an important signal that stimulates their migration, invasion, and metastases [102, 106]. In this context, succinate is considered an oncometabolite [107]. Many dysregulations of SDH in cancer cells are discussed [102].

Succinate accumulation and excretion is a hallmark of cancer cells, regardless of the presence or absence of mutations in SDH [108]. The factors responsible for the accumulation of succinate in cancer cells are summarized as follows [108]: (i) mutations in SDH; (ii) TRAP1—a key protein responsible for inhibiting SDH; TRAP1 is a mitochondrial chaperone, which is highly expressed in many types of cancer cells; TRAP1 inhibits respiratory complex II to decrease SDH activity, thus leading to high concentrations of succinate; (iii) the glyoxylate shunt, converting isocitrate to succinate by the enzyme isocitrate lyase; and (iv) the degradation of glutamate.

To these factors can be added the reversibility of SDH catalyzed reaction due to the high Q10H_2_/Q10 ratio in hypoxia or overexpression of mitochondrial *β*-oxidation. In acute hypoxia, the SDH reaction has been proven to be reversed [109, 110] and hypoxia is the main cause of succinate accumulation.

Anaerobic metabolism and accumulation of succinate in hypoxic tissues are well established [109, 110]. In the hearts of turtles and rats, it was found how this hypoxic metabolism was associated with increased glycolysis, as well as with the consumption of aspartate and glutamate [111]. Undoubtedly, this is a reversal of the Krebs cycle in one segment, starting with oxaloacetate and ending with succinate. The other segment of the Krebs cycle, from *α*-ketoglutarate (*α*-KG) to succinate, is in forward direction. According to author's opinion the forward segment can generate sufficient substrate levels of ATP and NADH to support oxaloacetate hydrogenation and succinate accumulation.

An interesting aspect of the ischemia-reoxygenation phenomenon is the time distribution of the two main events: (i) the accumulation of succinate and (ii) the reverse electron transport through complex I. Succinate accumulates during hypoxia, while RET occurs during reoxygenation and is the result of assimilation of accumulated succinate [110]. For RET to occur through complex I during ischemia-reoxygenation, the Q-pool must be maintained strongly reduced, to provide electrons for generating almost maximal protonmotive force (Δ*p*) by complexes III and IV, while also donating electrons for reverse transport through complex I. It takes about 20-25 minutes for ischemia to accumulate enough succinate, which is needed for significant ischemia-reoxygenation injury to the mouse heart [110].

**Figure 9:**
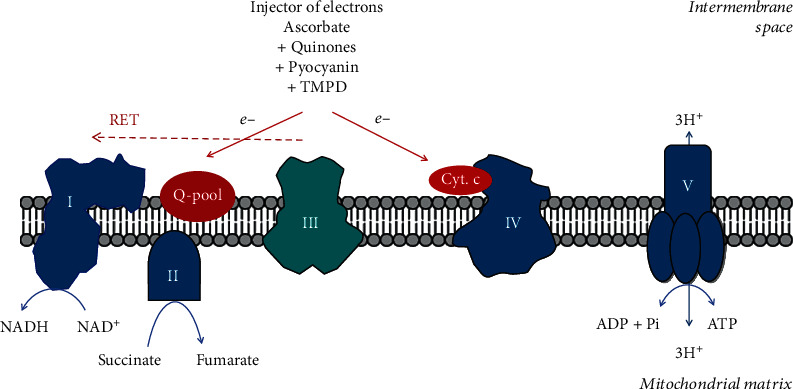
Schematic representation of the “reverse electron transport” (RET) in mitochondria (adapted according to Chance and Hollunger [112] and Warshaw et al. [113]). Mitochondria could synthesize NADH from NAD^+^ when there is enough ATP in the system plus some substrate that could inject electrons into the electron-transport chain, such as succinate, ascorbate, quinones, tetramethyl-phenylenediamine (TMPD), pyocyanin, and other artificial electron donors.

In fact, the RET phenomenon in isolated mitochondria has been well known since the 1960s under the name “energy-dependent reversal of respiratory electron flow” in mitochondria [112, 113]. In these early studies, it was described that mitochondria could synthesize NADH from NAD^+^ when there is enough ATP in the system plus some substrate that could inject electrons into the ETC. Such a substrate could be a succinate or an artificial reducing agent, such as the combinations of ascorbate with quinones or TMPD (Figure 9). Currently, it is generally accepted that NADH is synthesized at complex I when it operates in reverse mode. However, in the early studies, the RET phenomenon was not well explained as a mechanism, and it was not clear why this “energy-dependent reversal of respiratory electron flow” occurs only in the presence of ATP in the system. How does ATP energy drive this otherwise energetically unfavorable process? Today, this can be explained by the existence of mitochondrial potential in the mitochondria of mitochondrial DNA- (mtDNA-) free cells—the so-called *ρ*_*o*_ cells [114]. These cells do not have a functional ETC to maintain the mitochondrial potential. In them, ATP synthase works in the opposite direction and thus maintains the mitochondrial potential [114]. ATP synthase also functions in a reverse direction when the electrochemical potential becomes insufficient [115].

The phosphorylation activity of complex V could be modulated by a mitochondrial ATPase inhibitory factor (ATPIF) [116–118]. When mitochondrial electron transport is interrupted, complex V changes its mode of operation, transferring protons from the matrix to the intermembrane space. This process maintains Δ*p*, preventing cell death, but consuming ATP. ATPIF inhibits this reverse transfer of protons and thus prevents ATP depletion [117]. It is assumed that ATPIF may bind to complex V under normal conditions (when the proton flow is from the intermembrane space to the matrix), causing ROS production [117].

On the other hand, the operation of complex I is obviously reversible when there is a sufficiently high transmembrane potential and Δ*p* [110]. This suggests that mitochondrial transmembrane potential may be a more important parameter in cell physiology than even ATP production by mitochondria. The reversibility of complex I and ATP synthase ensures the maintenance of membrane potential in all cases of cell physiology. Only a drastic drop in ATP levels and a drop in the production of Q10H_2_ can destroy mitochondrial potential.

Is it possible for *β*-oxidation, which also depends on FAD as succinate, to inject electrons for RET? Guarás et al. found that the shift from glucose to fatty acids as a fuel increases electron flux through FAD-dependent enzymes, which saturates the Q-pool and leads to RET through complex I [97]. However, Schönfeld et al. did not detect RET-related ROS production and NADH accumulation, but they found a similar increase in mitochondrial potential, as well as in mitochondrial respiration by fatty acids and other substrates [119]. This was explained by the lack of supercomplex formation between complex I and the electron transferring flavoprotein, which is involved in the mFAO. Instead, supercomplex formation was found between complexes II and I and complexes II and III, which is thought to be related to RET. Despite limited experimental data in this field, the ability of *β*-oxidation to provide electrons to the ETC by FAD-dependent enzymes, for forward or backward electron flow, must be considered [118, 119]. In ischemia-reoxygenation phenomenon, increased Q10H_2_/Q10 ratio by *β*-oxidation may support succinate accumulation at oxygen deprivation, or to support RET at reoxygenation, when succinate is accumulated.

Recently, Li et al. reported that stimulation of cardiomyocytes with palmitate increases mFAO, which leads to intracellular accumulation of succinate and release of succinate in the extracellular space [120]. Interestingly, the authors found that accumulated succinate induces a hypoxia-like condition via increased expression of HIF-1*α*, as well as impaired PDH activity via upregulation of pyruvate dehydrogenase kinase 4 (PDK4) expression. It should be noted that increased succinate has also been found in the blood of humans and mice with obesity [121, 122]. It is well known that in obese individuals, the level of free fatty acids in the blood is markedly high [123]. In turn, this fact seriously links overactivated *β*-oxidation to SDH inhibition.

### 6.3. Acetyl-CoA and Acetylation of Mitochondrial Proteins

The most important evidence that *β*-oxidation (if overactivated) can reach the threshold of mitochondria overload with metabolites such as Q10H_2_, NADH, and acetyl-CoA and succinate is the rapid increase of nonenzymatic acetylation of mitochondrial proteins in acute activation of *β*-oxidation even in noncancerous cells and tissues.

Corbet et al. described a very interesting observation: *β*-oxidation acetylates mitochondrial proteins when mFAO is activated irregularly in acidic pH-adapted cancer cells [52]. In this study, ACC2 was inhibited in these cancer cells and significant acetylation of mitochondrial proteins was found as a consequence. It originates from nonenzymatic acetylation caused by elevated levels of acetyl-CoA.

The huge acetylation of mitochondrial proteins at lysine residues was discovered about fifteen years ago [124]. Proteomics analysis identified 277 unique acetylation sites in 133 proteins derived from two fractions of mouse liver mitochondria: one from fed mice and the other from fasted mice. Among the acetylated proteins, 62% were identified in both fractions, 14% were specific to fed mice, and 24% were specific to fasted mice. Many of the acetylated mitochondrial proteins have been identified as energy metabolism proteins and NAD^+^-dependent dehydrogenases. Six of identified enzymes are involved in the Krebs cycle, 26 proteins in OXPHOS, 27 in *β*-oxidation or lipid metabolism, and 14 NAD^+^-dependent dehydrogenases. Recently, with the refinement of experimental design, 2193 acetylation sites have been identified to belong to 434 mitochondrial proteins [125].

Emphasis is placed on the fact that mitochondrial acetylation could be of great importance for mitochondrial metabolism and mitochondrial acetylases and deacetylases should be responsible for this process. Before the discovery of mitochondria-located acetylases, attention was focused on mitochondria-located deacetylases: SIRT3, SIRT4, and SIRT5 [125]. Investigating the phenomenon of calorie restriction, Hirschey et al. observed that fasting upregulates the expression of SIRT3 in the liver and brown adipose tissues [126].

Thus, through studies of SIRT3 targets, it was found that acetylation of mitochondrial proteins in most cases is associated with inhibition of their enzyme activities. Recently, Parodi-Rullán et al. have concluded that an important part of the role of SIRT3 is to regulate mitochondrial bioenergetics [127]. Some of the most important metabolic enzymes, whose activity is modulated by acetylation/deacetylation, are discussed in their review article. Most of them are inhibited by acetylation, some of them are activated in some specific tissues, and some acetylated enzymes do not change their activity. We will outline only the cases for which the published information is mostly unidirectional:

- Acetylation decreases PDH activity, and PDH deacetylation is regulated directly by SIRT3
- In SIRT3^−^/^−^ mice, 71% of the proteins involved in mFAO in hepatic mitochondria are highly acetylated. These mitochondria have higher mFAO intermediates, suggesting decreased mFAO levels. Among all acyl-CoA dehydrogenases, the role of SIRT3 in regulating LCAD activity has been extensively studied. Acetylation of LCAD decreases its activity by 75% and SIRT3 can deacetylate LCAD and increase its activity
- Acetylation of mitochondrial complex I decreases its activity

### 6.4. Does Mitochondrial Acetylation Have Any Metabolic Role?

The first studies on the acetylation of mitochondrial proteins describe a different profile of acetylated proteins in calorie restriction, compared to a normal diet [128]. At the same time, activation of SIRT3 is also observed in calorie restriction. In 2010, Hirschey et al. found that LCAD was acetylated in the fed state and was deacetylated during fasting in SIRT3-expressing mice [126]. The same experiment was performed on mitochondria isolated from SIRT3^−^/^−^ mice. In this case, LCAD was found to be relatively hyperacetylated in the fed state and not deacetylated during fasting. These data indicate that SIRT3 is required for deacetylation of LCAD during fasting.

Direct nonenzymatic acetylation of mitochondrial proteins is thought to occur. However, initially, researchers were reluctant to accept that nonenzymatic reactions could have a significant physiological role in cells [129]. In 2013, Wagner and Payne give the first evidence of the possibility of nonenzymatic mitochondrial acetylation [130]. In 2014, Pougovkina et al. prove that acetyl-CoA, generated by the mFAO in the liver, is necessary and sufficient to drive the hyperacetylation of mitochondrial proteins during food deprivation [131]. However, other studies do not confirm acetylation as a consequence of starvation or calorie restriction [132]. Weinert et al. clarify this discrepancy by finding that fasting leads to moderately increased acetylation of mitochondrial proteins in wild-type animals and this effect is reversed in animals with adipose triglyceride lipase (ATGL) deficiency [132]. ATGL deficiency is an indication of a lack of free fatty acids in these animals, which means that mitochondrial *β*-oxidation is not well expressed. However, in animals with SIRT3 deficiency, acetylation is significant and markedly high at SIRT3-targeted sites. The authors explain that the different conclusions in the different studies are most likely due to the different effects of acute starvation and prolonged calorie restriction [132]. Acute starvation increases the acetylation of mitochondrial proteins, while prolonged calorie restriction induces the expression of SIRT3. Thus, deacetylation of mitochondrial proteins is delayed due to delayed expression of SIRT3. The authors measured the stoichiometry of acetylation in SIRT3-targeted sites and SIRT3 nontargeted sites and concluded that the role of SIRT3 is mostly to suppress the acetylation of mitochondrial proteins. This conclusion opens a new discussion on the topic: Does acetylation, and in particular nonenzymatic acetylation, have any physiological role?

Since mitochondrial hyperacetylation can inhibit *β*-oxidation enzymes in acute mFAO expression, it can be assumed that nonenzymatic acetylation is the third-line feedback mechanism for stopping *β*-oxidation. However, acetylation results in inhibition of other key components that are important for mitochondrial function such as PDH and complex I. This can cause a serious mitochondrial dysfunction if mitochondrial sirtuins are not expressed simultaneously during fasting (calorie restriction). In this case, the acetylation of mitochondria is only a side effect and the role of mitochondrial sirtuins is limited to maintain proteins in active state [132]. An alternative hypothesis could be that acetylation helps to rearrange mitochondria to mFAO and different FADH_2_/NADH ratios, as it is in the case of regulation of complex I by reverse electron transport and ROS-dependent mechanism [97]. Thus, inhibition of PDH by acetylation could be just an addition to the Randle's mechanisms supporting the antagonism of mFAO versus glucose metabolism.

The latter statement is supported by the notion that it is very difficult to inhibit mFAO at the LCAD level. The first enzymatic step of mitochondrial *β*-oxidation is catalyzed by the ACADs: very long-chain acyl-CoA dehydrogenase (VLCAD), long-chain acyl-CoA dehydrogenase (LCAD), medium-chain acyl-CoA dehydrogenase (MCAD), short-chain acyl-CoA dehydrogenase (SCAD), and three additional acyl-CoA dehydrogenases 9, 10, and 11 (ACAD9, ACAD10, and ACAD11) [133, 134]. Many review articles, referred to the study of Hischey et al. [126], state that acetylation inhibits mFAO by modulating LCAD activity [127, 129]. The substrate specificity of LCAD overlaps with that of VLCAD and LCAD9. Studies show that LCAD is difficult to detect in human tissues such as the liver, heart, and skeletal muscles [135, 136]. The specificity of LCAD is to unsaturated fatty acids such as oleic acid and branched-chain acyl-CoA substrates.

Inhibition of the mitochondrial TFP in the different subunits may lead to inhibition of further steps of *β*-oxidation. However, as described below, acetylation of this enzyme could arise from the function of additional acetylases, which may interfere with the nonenzymatic acetylation and the activation or inhibition of *β*-oxidation. These details seem very important, and no hasty conclusions should be drawn about the inhibition of mFAO in general by inhibiting a single *β*-oxidation enzyme from one type of acetylation.

It is interesting to note that hyperacetylation of mitochondrial proteins may occur due to increased glucose consumption in some cell types, such as insulin-secreting pancreatic *β*-cells. High-glucose concentrations have been found to increase acetylation of the TFP subunit alpha (encoded by the HADHA gene) and decrease fatty acid *β*-oxidation of islets [137]. On the other hand, SIRT3 has been found to deacetylate mitochondrial TFP subunit alpha and increase *β*-oxidation of fatty acids in pancreatic *β*-cells [137]. It should be noted that insulin secretion is significantly increased in cells with acetylated mitochondria. The same significant augmentation in insulin secretion was caused by 24-hour pretreatment of cells with palmitate. Overexpression of SIRT3 decreased basal insulin hypersecretion in rat islets pretreated with palmitate [137]. Palmitate-stimulated insulin secretion was markedly increased in islets of SIRT3-knockout mice, which was eliminated by SIRT3 overexpression. These results suggest that mitochondrial acetylation inhibits mFAO in pancreatic *β*-cells. In the presence of glucose, SIRT3 should be inactive, and glucose-induced acetylation inhibits mFAO and stimulates insulin secretion. However, in the absence of glucose, SIRT3 is activated, acetylation does not occur, mFAO is the main fuel to produce ATP, and insulin is not secreted. These experiments arise interesting questions: What happens if wild animals are on a diet high in fat and sugar, such as our usual daily diet? Will SIRT3 be expressed under these conditions?

Acetyl-CoA, required for the acetylation of proteins, can originate from pyruvate or fatty acids. The inhibition of PDH by acetyl-CoA, derived from pyruvate, can be interpreted as a feedback mechanism to limit glucose consumption in mitochondria. If glucose is the only fuel available to cells, this mechanism seems perfect. Excess of glucose causes acetylation of PDH and decreased glucose combustion in the Krebs cycle. NADH production in the Krebs cycle decreases consequently, and the increased NAD^+^ level activates mitochondrial sirtuins that deacetylate PDH. However, in pancreatic *β*-cells, the sensitivity of PDH to acetylation should be very low, the sensitivity of ECHA can be high, so glucose catabolism antagonizes fatty acid catabolism.

In most other cellular and tissue types, the nonenzymatic acetylation of PDH and decreased PDH activity appear to be the most pronounced effect in mitochondria. SIRT3, when is downregulated in these cell types, could not restore PDH activity when glucose is available (as in the fed state) [138]. SIRT3 deficiency leads to lactate production and causes loss of metabolic flexibility [138]. Upon nutrient deprivation, the expression and activity of pyruvate dehydrogenase kinase increase, which inhibits PDH by phosphorylation [139] (Figure 4). This process is reversible, and in fed state, pyruvate dehydrogenase phosphatases restore PDH activity. However, upon nutrient deprivation, mFAO is activated and acetylation of PDH further inhibits PDH activity and this is irreversible if SIRT3 is not activated. Acetylation of PDH could be nonenzymatic or enzymatically mediated by acetyl-CoA acetyltransferase (ACAT1) [140]. In both cases, it is caused by elevated concentrations of acetyl-CoA in mitochondria, provided by the increased mFAO. This example supports the idea that mitochondrial acetylation has the function of fine-tuning for the metabolic readjustment of mitochondria. In some cells, such as *β*-cells of the pancreas, PDH activity may not be sensitive to acetylation, but the enzymatic activities of mFAO may be sensitive. In most other types of cells and tissues (e.g., muscles), the opposite situation can be observed. If the production of acetyl-CoA originates from fatty acids and PDH activity is sensitive to acetylation, this mechanism could lead to antagonism of fatty acids versus the conversion of pyruvate to acetyl-CoA. If *β*-oxidation is overactivated by compromised AMPK-ACC2 mechanism, this could lead to accumulation of acetyl-CoA, inhibition of the pyruvate combustion, and generating a Warburg effect. Therefore, the AMPK-ACC2 mechanism in normal metabolism stops mFAO when ATP recovers and glucose is available. Inhibition of ACC2 by malonyl-CoA is very important to the cells: (i) when the increased cytoplasmic acetyl-CoA originates from fatty acid combustion, then the produced malonyl-CoA inhibits *β*-oxidation and FAS is not allowed due to lack of precursors and (ii) when the increased cytoplasmic acetyl-CoA originates from pyruvate, then FAS is allowed, and the produced malonyl-CoA inhibits *β*-oxidation. Thus, the combustion of glucose could be overactivated, and this will only lead to increased fatty acid synthesis, but *β*-oxidation cannot be overactivated if the AMPK-ACC2 mechanism is well tuned. However, the transition from fatty acids to glucose as a fuel requires increased SIRT3 expression, to increase PDH activity when glucose is already available.

Therefore, the activation of mitochondrial sirtuins, especially SIRT3, is very important to protect the organism from certain pathologies [141, 142]. Endogenous regulators of SIRT3 have recently been described, including the best-known activator NAD^+^ [142]. Calorie restriction, fasting, and exercise are most closely associated with increased expression of sirtuins [141, 143, 144], but the molecular mechanism of this induction is unclear. Genetic control of SIRT3 expression may depend on estrogen-related receptor *α* or nuclear respiratory factor 2, but it is not clear how these factors can be activated by calorie restriction [145].

### 6.5. Does Acetylation Inhibit or Activate *β*-Oxidation?

Although the main conclusion from most publications is that *β*-oxidation decreases due to mitochondrial acetylation, there are publications that contradict this conclusion. Alrob et al. investigated whether lysine acetylation controls cardiac glucose and fatty acid oxidation in obese and SIRT3-knockout mice on a high-fat diet [146, 147]. The authors observed increased fatty acid oxidation in response to a high-fat diet associated with hyperacetylation of LCAD and *β*-hydroxyacyl-CoA dehydrogenase (*β*-HAD). They concluded that this effect was controlled in part by downregulation of SIRT3 at high-fat diet. These results are completely opposite to the initial findings of Hirschey et al. that acetylation decreases *β*-oxidation activity [126].

Recently, the relationship between acetylation of mitochondrial proteins and the metabolic consequences of this process has been much complicated by the discovery of two lysine acyltransferases that have been found to interfere with mitochondrial acetylation: ACAT1 and GCN5L1. Thus, contradicted data and conclusions about the effect of acetylation on mFAO could be partly explained by the existence of enzymatic acetylation.

In the study of Fan et al., ACAT1 was found to be involved in the acetylation of the mitochondrial pyruvate dehydrogenase E1 (encoded by the PDHA1 gene) and its activating phosphatase PDP1 [140]. The main function of ACAT1 is to synthesize acetoacetyl-CoA by condensing two molecules of acetyl-CoA as a precursor for the synthesis of ketone bodies. The protein acetylase activity of this enzyme has been demonstrated to further increase the dependence of cancer cells on glycolysis by decreasing PDH activity. ACAT1 is also important for cancer cell proliferation and tumor growth. Recently, elevated enzyme activity of ACAT1 in various human cancer cell lines has been reported [148]. However, it should be borne in mind that acetylation by ACAT1 depends on elevated levels of acetyl-CoA in mitochondria exactly like nonenzymatic acetylation and is not under regulatory control.

Another acetyltransferase GCN5L1, showing significant homology to prokaryotic acetyltransferase (GCN5), was also found to acetylate mitochondrial proteins [149]. GCN5L1 has been shown to promote acetylation of SIRT3 respiratory chain targets and reverses the general SIRT3 effects on mitochondrial protein acetylation, respiration, and bioenergetics. In 2018, Thapa et al. observed in human liver cancer cells (HepG2) that GCN5L1 is involved in the inhibition of *β*-oxidation by acetylation of TFP subunit alpha (encoded by HADHA gene) [150]. GCN5L1 knockdown in HepG2 cells decreases acetylation of TFP subunit alpha and increases the activities of mFAO enzymes. In GCN5L1-knockout mice, TFP subunit alpha is not hyperacetylated at specific lysine residues (Lys-350, Lys-383, and Lys-406). The authors observed that acetylation at these sites is significantly associated with increased enzyme activity. Mice with liver-specific deletion of GCN5L1 were protected from hepatic lipid accumulation following a chronic high-fat diet and did not exhibit hyperacetylation of TFP subunit alpha, compared to wild-type controls. This study demonstrates that acetylation of mitochondrial proteins is also regulated at the genetic level, and the enzymatic acetylation is equally important for mitochondrial functions and mitochondrial *β*-oxidation.

However, it can also be assumed that in different tissues, different combinations of enzymatic with nonenzymatic acetylation and different expression profiles of SIRT3 may lead to combinations with diverse effects on metabolic processes and the level of *β*-oxidation.

Unlike hepatocytes, enzymatic acetylation can activate mitochondrial *β*-oxidation in cardiomyocytes. The same authors discovered in the heart that increased long-chain and short-chain acyl-CoA dehydrogenase acetylation correlated with increased enzymatic activity, lysine acetylation potentiates fatty acid oxidation in the cardiomyocyte, and this modification was partially regulated by the GCN5L1 acetyltransferase activity [151]. Based on this study, the different effect of acetylation on cardiomyocytes can be explained by the different activity of *β*-oxidation enzymes, depending on the enzymatic acetylation, as proposed by the authors. The upregulation of *β*-oxidation in cardiac tissue should not be so harmful, as cardiomyocytes have a very high ATP demand and the NADH/NAD^+^ ratio in these cells is constantly low. As will be discussed later, mitochondrial *β*-oxidation itself should not be harmful to cells when the NADH/NAD^+^ ratio is low, as is the case with active cells with high ATP demand (such as cardiomyocytes). However, if the NADH/NAD^+^ ratio increases, then *β*-oxidation could avoid the Krebs cycle, and this can lead to pathology.

In this context, the expression of *β*-oxidation combined with hypoxia can cause serious problems for the cells. It has long been clear that fatty acids are the most important respiratory substrate for the aerobic mammalian heart. However, under hypoxic conditions, free fatty acids can cause arrhythmias, increase the release of enzymes from myocytes, suppress myocardial contractility, and stimulate oxygen consumption over 15% by the aerobic heart [152]. These myocardial changes were observed in dogs and rats. Perfusion of hearts with a solution supplemented with fatty acids but containing a limited amount of oxygen (lowered to about 35%) to mimic hypoxia, leads to increased oxygen consumption and increased level of reduced pyridine nucleotides (NADH and NADPH) [152]. Moreover, the increased oxygen consumption is not related to ATP synthesis. The authors suggest that ATP production should be associated with a biosynthetic process or a “futile” cycle consuming ATP.

In addition, *β*-oxidation may increase the acute hypoxic effects. Li et al. found that treatment of cardiomyocytes with saturated fatty acid (palmitate) increases the oxidation of fatty acids, causes a hypoxia-like condition, and leads to the accumulation and excretion of succinate [120]. Inhibition of *β*-oxidation by trimetazidine prevents the accumulation of hypoxic succinate in cardiomyocytes and improves PDH activity.

Previously described destructive effects of *β*-oxidation are associated with the acute expression of hypoxia. In chronic hypoxia, an adaptive change occurs in cardiac mitochondria, leading to increased consumption of glucose and other substrates for energy needs, but not of fatty acids [153]. The reason is thought to be the high oxygen demand of the mFAO pathway. Maladaptive cardiac metabolism and cardiac dysfunction under chronic hypoxia were supposed to be the reason for pathology in these cases [153].

Based on the studies on the activation or inhibition of *β*-oxidation by enzymatic acetylation, it can be concluded that mFAO expression could be regulated in a different way in different organs, depending on their metabolic needs. In all cells, mFAO should be inhibited when glucose is available and increased when glucose is low. However, under conditions of hypoxia or other effects that can increase NADH in the mitochondrial matrix, the overexpression of *β*-oxidation could lead to pathology. Overexpression of *β*-oxidation could be the result not only of a compromised AMPK-ACC2 mechanism. This could be due to the increased availability of fatty acids, which causes impaired transition to glucose combustion along with increased mitochondrial *β*-oxidation, as it is in the case of high-fat diet.

### 6.6. High-Fat Diet, *β*-Oxidation, and Deacetylation of Mitochondrial Proteins

Over the last decade, a high-fat diet has been shown to decrease SIRT3 expression, impair mitochondrial functions, and decrease reliance on glycolytic substrates. In 2007, Koves et al. found that obesity-related insulin resistance and high-fat diet are characterized in skeletal muscles by excessive *β*-oxidation and impaired transition to a carbohydrate substrate during the fasting-to-diet transition [154]. The authors report that factors, suppressing the import of fatty acids in mitochondria, protect against lipid-induced insulin resistance. In 2011, Choudhury et al. subjected mice to chronic high-fat diet and observed decreased SIRT3 activity in the liver and a 3-fold decrease in hepatic NAD^+^ levels [155]. The authors identified 193 proteins that were preferably acetylated in mice on a high-fat diet compared to controls (on normal diet). SIRT3-deficient mice demonstrated even greater hyperacetylation of gluconeogenic and mitochondrial proteins in a high-fat diet. In addition to increased acetylation, SIRT3-deficient mice exhibited disruption of mitochondrial complexes II, III, and V. In 2015, Lantier et al. showed that muscles in SIRT3-deficient mice exhibit profound mitochondrial dysfunction with decreased reliance on glycolytic substrates and increased reliance on fatty acid substrates [156]. The authors observed that respiration decreased in the muscle fibers of SIRT3-deficient mice when malate-glutamate substrate was used, but oxygen consumption was significantly higher with malate palmitoyl-carnitine substrate. These studies are in consistent with the idea that fatty acid catabolism could antagonize glucose catabolism by acetylating mitochondrial proteins. This is a further development of the Randle's postulates [82], who stated that the provision of lipid fuel promotes *β*-oxidation and suppresses the glycolysis and oxidation of pyruvate due to inhibition of hexokinase, phosphofructokinase, and pyruvate dehydrogenase. Decreased SIRT3 expression in high availability of fatty acids may cause a permanent dependence of cells on fatty acids as a fuel and compromised transition of their metabolism to glucose combustion.

Over the last decade, it has also been reported that the antagonism between fatty acid catabolism and glucose catabolism in a high-fat diet does not depend only on decreased SIRT3 expression and/or activity. The acylcarnitine system has been found to be involved in the acetylation of mitochondrial proteins and in the antagonism of fatty acids versus glucose. Koves et al. emphasize that acylcarnitines may play a role in insulin resistance, and acylcarnitine production is considered a detoxifying system that allows mitochondrial efflux of excess acyl-groups [154]. This suggestion is based on their own data and the Ramsay's study [157]. Ramsay proposed that the role of the carnitine system is to maintain homoeostasis in the acyl-CoA pools of the cell, keeping the acyl-CoA/CoA pool constant even under conditions of very high acyl-CoA turnover [157]. Recently, Davies et al. demonstrated that carnitine acetyltransferase (CrAT) could be responsible for elevated levels of acetyl-CoA and acetylation of mitochondrial proteins, which is associated with metabolic dysfunction [158]. CrAT is an enzyme that buffers the mitochondrial pool of acetyl-CoA by converting short-chain acyl-CoAs to their membrane-permeable acylcarnitine analogues. CrAT-deficiency increases tissue acetyl-CoA levels and susceptibility to diet-induced lysine acetylation of broad-spectrum mitochondrial proteins, which is accompanied by decreased whole-body glucose control [158]. CrAT was found to be responsible for the export of excess acetyl-CoA from the mitochondrial matrix, and CrAT^−^/^−^ mice showed a similar overacetylated phenotype as SIRT3-knockout mice.

### 6.7. SIRT3: Pro- or Anticarcinogenic Factor in the Context of Antagonism between Fatty Acids and Glucose?

SIRT3 deacetylates PDH and thus increases aerobic glycolysis. It also increases OXPHOS by deacetylation of complex I [127], causing a return to normal noncancerous metabolism. This rationale leads some authors to suggest tumor suppressor functions of SIRT3. Kim et al. supported this hypothesis based on evidence of increased carcinogenesis in SIRT3-deficient cells and mice [159]. Fan et al. also supported the hypothesis based on indirect data showing that mitochondrial acetylase ACAT1 antagonizes with SIRT3 and ACAT1 is important for cancer cell proliferation and tumor growth [140]. Xiao et al. demonstrated induction of apoptosis upon overexpression of SIRT3 in lung adenocarcinoma cells [160].

In contrast, SIRT3 plays the role of an oncogene in some cancers. For example, in a study of Ma et al., which is very indicative, the basal level of oxygen consumption is relatively high in SIRT3-overexpressing acute myeloid leukemia cells, while SIRT3 knockdown shows the lowest oxygen consumption [161]. Moreover, the level of glycolysis is lowest in SIRT3-overexpressing cells derived from AML and highest in SIRT3 knockdown cells. High OXPHOS status in AML cells is associated with chemoresistance of these cells. Such data lead some authors to conclude that SIRT3 is an oncogene in cancers that are addicted to OXPHOS, and conversely, SIRT3 is a tumor suppressor in glycolysis-dependent cancers because it could activate glucose assimilation by mitochondria [142]. In our opinion, glycolysis and OXPHOS should not be opposed, as OXPHOS may coexist with anaerobic glycolysis when mitochondrial *β*-oxidation is overactivated.

In general, we should not expect cancer cells to return to normal metabolism. SIRT3 deficiency should increase carcinogenesis in the early stages of transformation. Overexpression of SIRT3 could increase oxidative phosphorylation, as well as *β*-oxidation and pyruvate as far as possible. However, this does not mean that normal expression of the Krebs cycle and normal NADH/NAD^+^ ratio could be recovered in already transformed cells. In addition, the pro- and anticancer effects of SIRT3 could be associated with deacetylation of other targets of mitochondrial or nonmitochondrial origin that are not related to PDH and/or ETC. A recent review article described the mechanisms by which SIRT3 exhibits its anticancer or prooncogenic effects [142]. It should be noted that most of the described anticancer effects of SIRT3 are not connected to direct deacetylation of PDH and complex I. Xiao et al. established that SIRT3-overexpressed lung adenocarcinoma cells undergo apoptosis [160]. However, lactate, OXPHOS, mFAO levels, and NADH/NAD^+^ ratio should be examined simultaneously to determine whether the effect is due to normalized mitochondrial function or depends on additional acetylated factors that are not connected directly to mitochondrial metabolism.

A similar case was observed with acetylation of p53. Ahmed et al. investigated the cross-link between p53 and SIRT3 in mice on a normal diet and a high-fat diet and their effect on survival and carcinogenesis [162]. The authors found that p53 expression significantly increased the lifespan of mice on a high-fat diet and decreased carcinogenesis, while SIRT3 expression attenuated its effect. Thus, the authors concluded that SIRT3 plays an oncogenic role and promotes carcinogenesis, induced by a high-fat diet in p53-expressing mice. However, Xiong et al. reported that SIRT3 deacetylates p53 and inactivates it via the ubiquitin-proteasome pathway [163]. Therefore, SIRT3 can be considered an antagonist of the high-fat diet. It can be concluded that high-fat diet leads to acetylation of p53, its stabilization, increases lifespan, and decreases carcinogenesis, while SIRT3 inactivates p53 and plays an oncogenic role. Thus, the oncogenic role of SIRT3, induced by a high-fat diet, does not depend on the denormalization of mitochondrial function. The role of a high-fat diet in increasing lifespan by stabilizing p53 explains the pathologies induced by this diet. The downregulation of SIRT3 in a high-fat diet should be beneficial for the cells due to the stabilization of p53. Decreased SIRT3 expression at high-fat diet may not be so harmful for cells. While the Krebs cycle and ETC are not impaired and ATP demand is high, cells will simply increase energy production at the expense of mFAO. However, hypoxia or dysfunctions of ETC and Krebs cycle combined with overactivated *β*-oxidation and decreased SIRT3 expression can lead to pathology.

Complex I is among the many proteins found to be nonenzymatically acetylated in the mitochondria of acidic pH-adapted cancer cells [52]. The acetylation of complex I was suppressed by pretreatment with a CPT1 inhibitor, the regulatory enzyme of mFAO [52] (Figure 3). Both circumstances (decreased activity of complex I and overactivated *β*-oxidation) lead to an increased NADH/NAD^+^ ratio and this ratio can reach the point of inhibition of the Krebs cycle. Acetylation coexists with deacetylation, which is mainly due to mitochondrial SIRT3 deacetylase. Therefore, the acetylation state of proteins is а matter of balance. There is also acetylation of many other mitochondrial proteins, but in the specific case associated with complex I, it seems that SIRT3 deacetylase cannot be activated if the concentration of NAD^+^ is low in the mitochondria. It has recently been found that downregulation of complex I leads to decreased SIRT3 activity by decreasing the NAD^+^/NADH ratio [164]. Given that the expression of SIRT3 is delayed after activation of *β*-oxidation, this is a rather insurmountable situation in which NADH should accumulate in the mitochondrial matrix, as emphasized in earlier works of Garland et al. and Randle et al. [83, 84]. As already mentioned above, Guarás et al. found that electron flux through the FAD-dependent pathway (via fatty acids or complex II) downregulates the content of complex I to adjust the electron flux from a different FADH_2_/NADH ratio [97]. This means that getting out of the *β*-oxidation trap is quite difficult and it is necessary to know the limit to which the NADH/NAD^+^ ratio in the mitochondrial matrix can increase.

Summarizing, when mitochondrial *β*-oxidation is activated simultaneously with decreased SIRT3 activity and/or decreased acylcarnitine system capacity, it can inhibit PDH and complex I by acetylation and other mechanisms. However, *β*-oxidation appears to be more dependent on regulation by acetylases, which in turn are regulated by other factors. The maladaptive return to pyruvate consumption, following exposures such as hypoxia and a high-fat diet, may be responsible for permanent hyperacetylation of PDH and complex I. Inhibition of complex I can alter the redox state of mitochondria in favor of increased NADH/NAD^+^ ratio. Because the mitochondrial TFP, catalyzing *β*-oxidation, is less sensitive to NADH feedback inhibition, continuous expression of *β*-oxidation seems highly possible. In this case, activation of mitochondrial sirtuins appears less possible and *β*-oxidation may continue to operate under conditions of inhibited *α*-KGDH and NAD^+^-dependent IDH of the Krebs cycle. This situation could activate the citrate-malate shuttle instead of the Krebs cycle and *β*-oxidation will support cataplerosis instead of OXPHOS. We emphasize the cataplerosis as the most important event in the context of carcinogenesis. The mechanism of activation of citrate export has been found to be part of the normal functioning of immune cells when they are activated. The mitochondrial citrate/malate exchanger (citrate/isocitrate carrier, CIC) is a target of acetylation, and the level of acetylation is higher in glucose-deprived medium than in normal glucose medium [165]. Acetylation of CIC has been shown to result in greater citrate efflux from mitochondria in exchange for malate [165]. In addition, cytoplasmic isocitrate dehydrogenase IDH1 is increased in activated immune cells. IDH1 catalyzed synthesis of NADPH, which could be part of the citrate-isocitrate NADPH exporting shuttle, also easily activated upon mFAO activation (see Section 7.2).

## 7. Reductive Carboxylation as a Consequence of Impaired Redox State in Mitochondria: NADH/NAD^+^ and NADPH/NADP^+^ Ratios

How could *β*-oxidation be involved in the regulation of the NADPH/NADP^+^ redox state?

**Figure 10:**
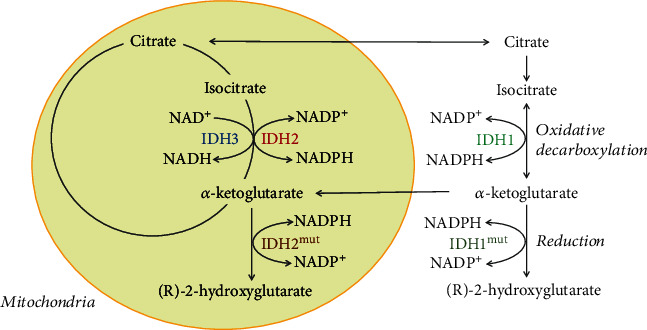
Subcellular localization and chemical reactions catalyzed by wild-type isocitrate dehydrogenase isoenzymes (IDH1, IDH2) and tumor-derived isocitrate dehydrogenase mutant isoenzymes (IDH1^mut^, IDH2^mut^) (according to Tommasini-Ghelfi et al. [179]).

The answer may exist in the unusual role of AMPK, discovered in 2012 by Jeon et al. [166]. The authors found that in metabolic stress, especially in glucose deficiency, AMPK activation is involved in maintaining the redox state of NADPH/NADP^+^. At a low-glucose supply, PPP does not provide sufficient amount of NADPH. Subsequent activation of AMPK inhibits ACC1 and ACC2, leading to decreased NADPH consumption by FAS and increased NADPH production by mFAO (Figures 3(b) and 10). This determines the role of AMPK not only in restoring ATP levels but also in restoring NADPH/NADP^+^ redox state during glucose deficiency.

Mitochondrial nicotinamide nucleotide transhydrogenase (NNT) is a major part of this function. This enzyme transfers reducing equivalents from NADH to NADPH. NNT is located on the inner mitochondrial membrane and is energy dependent. It is driven by the mitochondrial proton motive force (Δ*p*) and its activity is directly dependent on the respiratory state of the mitochondria [167]. In the liver mitochondria of mice, the contribution of NNT to NADPH production has been found to vary between 0 and 100% [167]. The proton motive force makes NNT able to increase NADPH/NADP^+^ ratio more than 500 times compared to the NADH/NAD^+^ ratio, as well as NADPH amount more than 95% in the mitochondrial matrix [167, 168]. The significance of NNT overexpression for cancer progression has been described in several studies [169–171]. Given the relatively high amount of NADH in the cancer cells [172], as well as the higher Δ*p* of the cancerous mitochondria [173], it can be assumed that the NADPH levels in cancerous mitochondria are extremely high. However, there is a shuttle that is activated by high concentrations of NADPH to export these reducing equivalents from mitochondria into the cytoplasm (see below).

In cancer cells, the NADH/NAD^+^ ratio is significantly higher than in normal cells [172], and its further increase depends mainly on inhibition of LDH rather than inhibition of complex I. In contrast, in noncancer cells, the increase of NADH/NAD^+^ ratio depends mainly on the inhibition of complex I (e.g., with rotenone) rather than on the inhibition of LDH or malate-aspartate shuttle. In addition, inhibition of LDH in normal cells does not increase NADH levels in mitochondria [172]. LDH expression could be considered a mechanism for decreasing the NADH/NAD^+^ ratio in the cytoplasm, which helps cancer cells to decrease the same ratio in mitochondria through the malate-aspartate shuttle. The study of Sullivan et al. supports this assumption [174]. The authors found that ETC inhibitors suppressed the proliferation of cancer cells by interrupting the synthesis of aspartate. The addition of aspartate to cells with inhibited ETC restores their proliferation. Inhibition of aspartate synthesis is caused by NAD^+^ deficiency. In the same study, it was clearly demonstrated that electron acceptor deficiency limits the proliferation of respiratory-deficient cancer cells. The addition of any electron acceptor that produces NAD^+^, such as pyruvate or *α*-ketobutyrate, potentiates proliferation through the production of NAD^+^. These studies undoubtedly suggest that cancer cells have an increased NADH/NAD^+^ ratio compared to normal cells [172, 174]. In normal cells, this ratio is independent of LDH, because it is lower and still depends on the inhibition of ETC. In cancer cells with inhibited ETC, the NADH/NAD^+^ ratio is close to the maximum, and further inhibition of ETC does not increase this ratio in mitochondria, but directly affects lactate-pyruvate balance. This should be the reason why cancer cells depend on compensatory processes that regulate the redox state such as LDH-dependent equilibrium. Surprisingly, additional damage of the cancerous ETC leads to inhibition of aspartate synthesis and the inability of cancer cells to synthesize purines, pyrimidines, and DNA, respectively [174]. The latest finding shows that mitochondrial function, associated with ATP synthesis, may not be so crucial for cancer cell survival. Their survival depends on another mitochondrial function associated with aspartate synthesis. This aspect is discussed in Section 8.3 of the article.

In general, decreased NADH utilization, as well as increased NADH production, should be the reason for the increased NADH/NAD^+^ ratio. Increased NADH production is required to maintain mitochondrial potential. However, increased NADH production in the matrix by NAD-dependent IDH and *α*-KGDH at the elevated background NADH levels seems unlikely and NADH production could be most easily achieved by increased *β*-oxidation.

### 7.1. Reductive Carboxylation Is Driven by the High NADPH/NADP^+^ Ratio

Defects in complex I and complex III directly support the reductive carboxylation against the normal direction of the Krebs cycle. Gameiro et al. noted that the sources of NADPH contribution to reductive carboxylation in mitochondria are unknown, but the authors found that the NNT enzyme is an important intermediate of the driving force that causes reductive glutaminolysis [175]. Other authors have demonstrated that cancer cells with defective mitochondria use glutamine-dependent reductive carboxylation rather than oxidative metabolism as the major pathway for citrate formation [176]. They also show that the reductive glutamine-dependent pathway is dominant mode in the metabolism of renal carcinoma cells derived from patient with mutations in fumarate hydratase, as well as in cells with normal mitochondria subjected to acute pharmacological inhibition of ETC [176]. They assume that the cause and driving force of this mechanism is the increased NADH/NAD^+^ ratio in the mitochondrial matrix because of the decreased oxidative capacity of the Krebs cycle. According to these authors, the NADH/NAD^+^ ratio could be partially dissipated by NAD(P)-transhydrogenase, which transfers reducing equivalents from NADH to NADPH and in turn can cause NADPH-dependent reductive carboxylation by isocitrate dehydrogenases (IDH1 and IDH2). It has been found in hypoxic melanoma cells that the reverse flux of the Krebs cycle through IDH1 and IDH2 is also required for lipogenesis and viability [177]. This is a good model to explain the mechanism of reductive carboxylation, but it is incomplete. It should also be born in mind that inhibition of ETC may increase the NADH/NAD^+^ ratio for a short time and may decrease the oxidative capacity of the Krebs cycle. This means that the Krebs cycle is inhibited and cannot produce NADH anymore. Therefore, we need to look for other processes that can produce NADH.

### 7.2. Role of the Isocitrate-*α*-Ketoglutarate Shuttle in the Export of NADPH from Mitochondria

The inner mitochondrial membrane is impermeable to NAD(P)H and the exchange between cytosolic and mitochondrial NAD(P)H pools is conducted by the isocitrate-*α*-KG shuttle [178]. This NADPH shuttle functions through the isoenzymes IDH1 and IDH2 (Figure 10). In the mitochondrial matrix, NADP^+^-dependent IDH2 converts *α*-KG into isocitrate by oxidizing NADPH to NADP^+^. The isocitrate is pumped into the cytosol in exchange for malate by the citrate carrier protein (encoded by the SLC25A1 gene). In the cytosol, IDH1 catalyzes the reverse reaction by transforming isocitrate to *α*-KG and NADP^+^ to NADPH. Subsequently, *α*-KG is transported in the mitochondrial matrix by the *α*-KG/malate antiporter as a carrier in the malate-aspartate shuttle. Thus, the isocitrate-*α*-KG shuttle plays a pivotal role in maintaining cellular levels of NADPH [178].

### 7.3. Dependence of Cancer Cells on the Citrate-Malate Shuttle

**Figure 11:**
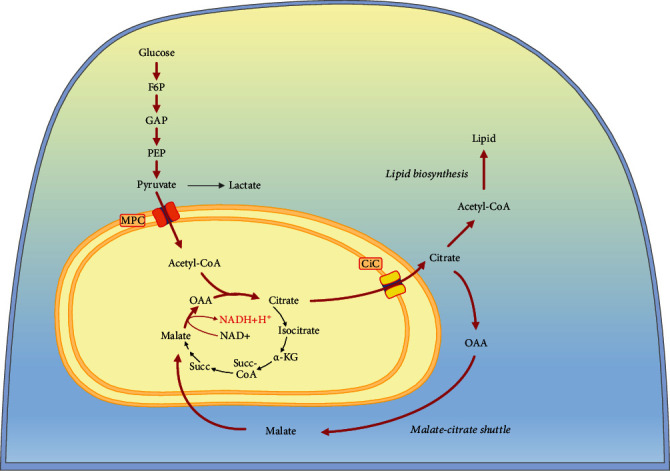
Schematic representation of increased function of the citrate-malate shuttle in hepatocellular carcinoma, according to Lei et al. [180].

Recently, Lei et al. have used metabolomic and transcriptomic analyses to detect differences in metabolic pathways between normal, precancerous, and hepatocellular carcinoma (HCC) in the liver [180]. The authors found decreased catabolic activity of the Krebs cycle in HCC, as well as a significant increase in the levels of citrate, malate, and fumarate, which indicates enhanced function of the citrate-malate shuttle. Their conclusion is that the conversion of malate to fumarate is sufficient for the generation of electrons for complex I and for the synthesis of ATP. The consumption of pyruvate associated with this shuttle is considered crucial in the detoxification of lactate (Figure 11). However, the increase of aerobic glycolysis and lactate production in HCC is well established [181], which contradicts this conclusion. In addition, the lack of concomitant analysis of mFAO does not allow a comprehensive analysis of the metabolic features of HCC, given that hepatitis B virus derived HCC has been reported to depend on mFAO [182]. Inhibition of the Krebs cycle in HCC has been shown to depend on decreased expression of *α*-KGDH, one of the rate-limiting components of the key mitochondrial multienzyme *α*-KGDH complex [183]. In the same study, decreased levels of this key component of the Krebs cycle and increased *α*-KG/citrate ratio were considered a main cause of increased reductive carboxylation and reductive glutaminolysis. It should be noted that increased NADPH production was also found [180], and this is an important reason for the reductive glutaminolysis. Reductive glutaminolysis requires high mitochondrial NADPH and this could only be achieved through the work of NNT at the presence of additional amount of NADH. As already mentioned, increased NADH production in the mitochondrial matrix is most easily achieved by *β*-oxidation, especially when the Krebs cycle is inhibited. However, *β*-oxidation was not examined in this study [180].

## 8. Definition of the “*β*-Oxidation Shuttle”

**Figure 12:**
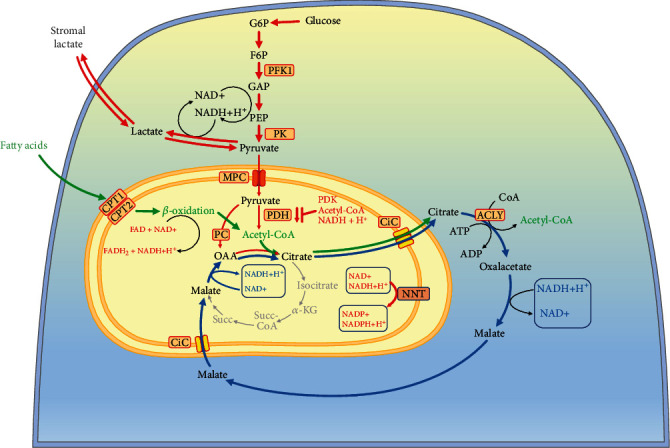
Schematic representation of the “*β*-oxidation shuttle” in mitochondria: connection of *β*-oxidation to the citrate-malate shuttle and formation of a separate and independent metabolic pathway, which has its own energy efficiency, own oxygen consumption, and own influence on other metabolic pathways. The green arrows indicate the metabolic flux from fatty acids. The red arrows indicate the metabolic flux from glucose. The red blunt ends indicate the inhibition of a particular enzyme. The blue arrows indicate the malate-citrate shuttle. ACLY: ATP citrate lyase; CIC: mitochondrial citrate carrier; CTP1 and CTP2: carnitine palmitoyl transferases 1 and 2; F6P: fructose-6-phosphate/fructose-1,6-bisphosphate; G6P: glucose-6-phosphate; GAP: glyceraldehyde-3-phosphate; a-KG: a-ketoglutarate; MPC: mitochondrial pyruvate carrier; NNT: NAD(P) transhydrogenase; OAA: oxaloacetate; PDK: pyruvate dehydrogenase kinase; PDH: pyruvate dehydrogenase; PC: pyruvate carboxylase; PFK1: phosphofructokinase-1; PK: pyruvate kinase; PEP: phosphoenolpyruvate; Succ: succinate.

When *β*-oxidation is overactivated by impaired AMPK-ACC2 mechanism or high availability of fatty acids, it may change the redox state of mitochondria in favor of an increased NADH/NAD^+^ ratio, because the TFP is less sensitive to inhibition by a NADH-dependent feedback mechanism. If the ATP demand of the cell is low, it will further alter the reduced state of the mitochondrial matrix and the increased NADH. Effects such as ETC dysfunctions and partial hypoxia, capable of changing the redox balance of the mitochondrial matrix in the direction of a high NADH/NAD^+^ ratio, could also be combined with overactivated mitochondrial *β*-oxidation and could inhibit certain enzymes of the Krebs cycle. Mutations in some enzymes of the Krebs cycle (common in many cancers) increase the ability of *β*-oxidation to be functional without part of the Krebs cycle. If *β*-oxidation is not connected with the Krebs cycle but to the citrate-malate shuttle, it forms a separate and independent metabolic pathway, which has its own energy efficiency, own oxygen consumption, and own influence on other metabolic pathways. We called this metabolic state “*β*-oxidation shuttle” to distinguish it from other metabolic states (Figure 12). The “*β*-oxidation shuttle” brings a huge advantage to the cell related to the export of citrate and NADPH from the mitochondria into the cytoplasm and supporting all anabolic processes.

The “*β*-oxidation shuttle” can maintain the NADH/NAD^+^ ratio at the highest possible level in the mitochondrial matrix. Dissipation of this reduced state occurs by transferring reducing equivalents from NADH to NADP^+^ via NNT and by exporting reducing equivalents such as NADPH from the mitochondrial matrix to the cytoplasm. LDH, which produces cytoplasmic NAD^+^, can dissipate this pressure by decreasing the import of cytoplasmic reducing equivalents into the matrix via the malate-aspartate shuttle [184]. Thus, activated LDH in cancer cells that are addicted to the “*β*-oxidation shuttle” should act as a compensatory mechanism to decrease the irregularly changed redox state of the mitochondrial matrix. Pyruvate could be eliminated from the metabolism by other reactions, for example, transamination into alanine, if pyruvate is not required for ATP production in mitochondria. Alanine could be easily excreted by cells and used by the liver in the glucose-alanine cycle. However, cancer cells prefer to get rid of excess lactate through LDH and instead to face the low pH. We assume that the goal of increased anaerobic glycolysis in the cell over the need for pyruvate in mitochondria is not only to produce ATP but also to compensate the redox state of the mitochondrial matrix through expression of LDH. Some types of cancer cells also produce additional pyruvate through the malic enzyme [185]. This may also be a compensatory mechanism for decreasing the NADH/NAD^+^ ratio in them. Additionally, malate dehydrogenase 1 (MDH1) which is part of this shuttle can activate glycolysis by producing NAD^+^ in the cytoplasm, as an alternative to LDH as a supplier of NAD^+^ in cancer cells [186].

We propose that the “*β*-oxidation shuttle” consists of a mitochondrial *β*-oxidation and a citrate-malate shuttle. In turn, the citrate-malate shuttle consists of a transmembrane transporter and several enzymes: mitochondrial CIC, ATP-citrate lyase (ACLY), and two malate dehydrogenases—MDH2 and MDH1.

What is the impact of all these components in the context of carcinogenesis?

### 8.1. Citrate/Isocitrate Carrier in Cancer

Citrate is at the crossroads of many metabolic pathways and is an indispensable source of carbons in the mitochondria and cytosol, a key substrate for the generation of energy, and an allosteric modulator of several enzymes as well (Figure 3). When glucose is abundant, the majority of intracellular citrate comes predominantly from the oxidative decarboxylation of pyruvate in the mitochondria. Then, it is exported to the cytoplasm via the citrate/isocitrate carrier in exchange for malate.

CIC is a nuclear-encoded protein located in the inner mitochondrial membrane. It plays an important role in lipogenesis and is also a key component of the isocitrate-oxaloacetate and the citrate-malate shuttles.

Numerous studies have proven the proinflammatory and prooncogenic effects of CIC, as well as its participation in reversing the Warburg effect. The fundamental role of CIC and its upregulation in cancer, inflammation, and beyond is well described in a recent review article by Mosaoa et al. [187]. CIC was found to be overexpressed in different types of cancer: breast cancer [188], colorectal cancer [189], prostate cancer [190], hepatomas [191], thyroid carcinoma [192], and others. These multiple studies clearly demonstrate that CIC is essential for the proliferation of different types of cancer cells. They also demonstrate the potential of CIC inhibitors in anticancer therapy and especially as a therapeutic strategy for specific targeting and elimination of therapy-resistant cells.

CIC is thought to support the cell proliferation by promoting OXPHOS and suppressing glycolysis, presumably due to the negative feedback loop that cytosolic citrate (provided by CIC) has on PFK1 [193, 194] (Figure 3(b)). How CIC affects OXPHOS is not yet entirely clear, but such regulation may occur at least in part due to the promotion of malate entry into mitochondria, which in turn leads to increased flux in the Krebs cycle and the generation of reducing equivalents (such as NADH) for ETC [187].

CIC expression, as assessed by the transcription rate of CIC promoter, was found to be controlled by the major regulators of lipid anabolic pathways, sterol regulatory element-binding factor 1 (SREBP1) [195], and of Forkhead Box A1 (FOXA1), which via CIC induces glucose-stimulated insulin secretion in *β*-cells of pancreas [196]. The tumor suppressor p53 also interects with the CIC promoter [197]. However, while wild-type p53 suppresses CIC transcription, tumor-associated mutants of p53 do not bind to the CIC promotor directly but are recruited therein through interaction with the transcription factor Forkhead Box O1 (FOXO1). FOXO1 has very important activities in the regulation of insulin signaling, gluconeogenesis, and glycogenolysis.

Various transcription factors have been found to interact with the CIC promoter and induce CIC expression in response to inflammation and proliferation: nuclear factor kappa B (NFkB), signal transducer and activator of transcription 1 (STAT1), Myc, HIF-1*α*, and others [198, 199]. These mechanisms of CIC activation suggest that this protein may provide a link between oncogenic pathways and glucose and lipid metabolism in cancer cells.

High levels of CIC in tumors are thought to allow adaptation to nutritional stress and resistance to mitochondrial respiration injury [193]. It is interesting to note that CIC expression is also regulated by diet. For example, starvation and a diet enriched with polyunsaturated fatty acid (PUFAs) could significantly downregulate the expression of this transporter protein [200]. Decreased expression of proteins involved in FAS, including CIC, is also relevant to the loss of adipose tissue mass in cancer-bearing animals subjected to chemotherapy [201].

### 8.2. ATP-Citrate Lyase in Cancer

ATP-citrate lyase is a key metabolic enzyme that catalyzes the generation of acetyl-CoA. ACLY is upregulated in cancer cells and is required for their growth [79]. ACLY overexpression has been associated with increased tumor progression in many cancers: breast, lung, brain, colorectal, hepatocellular, and others. ACLY expression and inhibition have been also associated with other chronic diseases such as diabetes, obesity, nonalcoholic fatty liver disease, cardiovascular diseases, inflammatory disorders, and neurodegenerative diseases [79].

Upregulation of ACLY could be one of the key events in malignant cell transformation. Icard et al. hypothesized that upregulation of ACLY is responsible for maintaining low cytosolic citrate levels in cancer cells, favoring enhancement of glycolysis (which would be inhibited by high citrate) and activation of oncogenic drivers, such as the PI3K/Akt and WNT/*β*-catenin pathway [202]. Indeed, citrate production may not be a key characteristic of cancer cells, but increased citrate utilization may be one of the key events in the transformation of cancer cells. For example, prostate epithelial cells produce large amounts of citrate by accumulating Zn^2+^ in mitochondria, leading to inhibition of mitochondrial aconitase (mtACON). In contrast, prostate cancer cells have lost their ability to accumulate Zn^2+^ and they have decreased citrate levels [202]. Based on this difference between normal epithelial and cancer cells, Costello and Franklin concluded that the transition of normal citrate-producing epithelial cells to malignant citrate-oxidizing cells is a major metabolic event in the carcinogenesis of prostate cancer. However, prostate cancer exhibits unique metabolism with high rates of de novo fatty acid synthesis driven by activation of the androgen receptor [203]. How do prostate cancer cells supply the cytoplasm with the amount of citrate needed for FAS, if the citrate is oxidized in the Krebs cycle?

It has been found that prostate cancer cells may depend on the reductive metabolism of glutamine when treated with metformin [204, 205]. If reductive synthesis of citrate is increased in prostate cancer cells, then it is clear why mtACON inhibition is lost in them. Aconitase is required for the reductive synthesis of citrate. In addition, based on many experimental observations, Liu summarized that “fatty acid oxidation is the dominant bioenergetic pathway in prostate cancer” [206]. It can be concluded that decreased level of citrate in the cytoplasm is due to its increased utilization by ACLY and FAS. The produced citrate does not participate in the production of ATP, but in the production of lipids. Therefore, overactivated mitochondrial *β*-oxidation should be tightly related to the reductive carboxylation of *α*-KG. Details about the relationship between the “*β*-oxidation shuttle” and the glutaminolysis pathways are described in Section 10. The last statement is an indication that the primary reason for the cancer cell transformation should be sought not only in the expression of *β*-oxidation shuttle, which exports citrate into the cytoplasm, but also in the overexpressed lipid synthesis pathways that consume the citrate.

A direct relationship has been established in the activity and expression of ACLY and CPT1 in cancer cells [207]. In early studies, the authors demonstrated that inhibition of ACLY induces apoptosis and/or suppression of growth in a subset of cancer cells [208]. They also observed that suppression of cell growth mediated by ACLY inhibition was associated with paradoxical lipid accumulation [209]. Interestingly, ACLY depletion regulates the expression of CPT1, which is a transporter of mitochondrial fatty acids [207]. We could suggest that the reason for the suppression of cell growth and the paradoxical accumulation of lipids is in the downregulation of the “*β*-oxidation shuttle”.

Acetylation has also been shown to stabilize ACLY and promote lipid biosynthesis and tumor growth [210].

Recently, Basappa et al. found that phosphoinositide 3-kinase (PI3K) and Src-family kinase (SFK) Lyn, that are constitutively activated in many cancers, can activate ACLY in cancer cells [211].

### 8.3. Malate Dehydrogenases MDH2 and MDH1 in Cancer

An important circumstance related to the “*β*-oxidation shuttle”, which has not been discussed so far, is its dependence on mitochondrial malate dehydrogenase 2. MDH2 is part of the Krebs cycle, and the catalyzed reaction is referred as reversible, although its standard Gibbs free energy is positive, and the backward reaction is preferred under standard conditions [101]. However, MDH2 is allosterically regulated by three metabolites—citrate, malate, and oxaloacetate [212]. These three metabolites inhibit MDH2 activity in backward direction from NADH to NAD^+^. They also inhibit MDH2 activity in forward direction from NAD^+^ to NADH, but only at low concentrations of malate and NAD^+^ as substrates. In contrast, citrate, malate, and oxaloacetate activate MDH2 in forward direction from NAD^+^ to NADH at higher concentrations of substrates. Based on a study of Sahlin and Katz, which reported that mitochondrial NADH at rest does not exceed 60% [88], it can be concluded that NAD^+^ in the mitochondrial matrix should be enough to prevent inhibition of the forward reaction. However, the presence of malate and citrate in the matrix should activate the enzyme in a forward direction.

This model of regulation of the enzyme activity of MDH2 also suggests that higher amounts of enzyme in the mitochondria will accelerate the forward reaction (in the direction of NADH) and will support the existence of the “*β*-oxidation shuttle” in cancer cells. Recently, Ma et al. found the level of urinary protein MDH2 to be higher in patients with non-small-cell lung cancer (NSCLC), compared to the same parameter in the healthy population [213]. MDH2 expression levels were also higher in lung cancer tissues than in normal tissues. MDH2 knockdown in lung cancer cell lines inhibited cell proliferation.

The presence of a high amount of mitochondrial citrate, required to activate the forward work of MDH2 in the mitochondrial matrix, should be beyond doubt in cancer cells. As already discussed, decreased citrate level in the cytoplasm is associated with increased expression of ACLY and FAS in cancer cells, but citrate production does not decrease due to increased glutaminolysis. Thus, the increased export of citrate for FAS should be a major consequence of high mitochondrial citrate.

The availability of malate should be increased by anaplerotic glutaminolysis, which is a main characteristic of cancer cells (see Figure S2 in the Supplementary Materials). The presence of malate required to activate the forward work of MDH2 in the matrix depends also on the work of cytoplasmic MDH1. MDH1 functions well in the direction of malate synthesis at the presence of high levels of cytoplasmic NADH. Increased anaerobic glycolysis of cancer cells should be a good source for NADH production, but increased LDH activity in cancer cells utilizes these reducing equivalents. However, LDH cannot decrease the NADH/NAD^+^ ratio below the level achieved by glycolysis. Recently, Hanse et al. demonstrated on various cancer cell lines, that cytosolic MDH1 is an alternative to LDH as a supplier of NAD^+^. The amplification of MDH1 occurs with an impressive frequency in human tumors and correlates with a poor prognosis [186]. Thus, MDH1 can accelerate glycolysis by producing NAD^+^. On the other hand, if additional processes producing NADH in cytoplasm are activated in cancer cells, they will increase the NADH/NAD^+^ ratio and MDH1 will decrease the additional reductive pressure in the cytoplasm. Cytosolic aldehyde dehydrogenases (ALDHs) are discussed as suppliers of cytoplasm with NADH [214]. The second important supplier of cytoplasm with NADH is the polyol pathway for the synthesis of fructose from glucose, which is overactivated in many types of cancer cells [215–217]. Both factors (ALDHs and polyol pathway) appear to be important for cancer cell survival. Thus, additional suppliers of the cytoplasm with reducing equivalents such as NADH could be interpreted as accelerators of MDH1 for malate production and for increasing the availability of malate in mitochondria, which could support mitochondrial MDH2.

The dependence of cancer cells on ALDHs was discovered by Kang et al. who found that cytosolic ALDHs were associated with non-small-cell lung cancer (NSCLC) [214]. The authors inhibited ATP synthesis up to 80% by combining an ALDH inhibitor (gossypol) and a complex I inhibitor (phenformin). Based on their studies, in 2018, Kim concluded that cancer cells, or at least NSCLC, use predominantly OXPHOS to synthesize ATP by using cytoplasmic NADH [218]. However, this general conclusion can be questioned, given that all influences, such as cytoplasmic manipulations of NADH, inhibition of the malate-aspartate shuttle, and inhibition of complex I, could lead to catastrophic change in the redox state of the NADH/NAD^+^ pair. The same author interpreted the inhibition of glutaminolysis as an inhibition of mitochondrial ATP synthesis. On the other hand, inhibition of glutaminolysis results in a deficiency of glutamate, which is required for GOT2 to synthesize aspartate. Sullivan et al. observed that changing the redox state by ETC inhibitors and other redox influences may affect aspartate synthesis. Cancer cells whose growth was suppressed by ETC inhibitors die from aspartate deficiency (not from ATP deficiency) and could be saved by the addition of aspartate [174]. The second notion is that ETC inhibitors could inhibit the malate-aspartate shuttle and increase cytoplasmic NADH/NAD^+^ ratio. The last can lead to inhibition of glycolysis and depletion of ATP. Mitochondrial function is important for the survival of cancer cells, but this does not mean that the production of mitochondrial ATP is the only vital factor.

MDH2 and cytoplasmic MDH1 are part of the citrate-malate shuttle, but they are also part of the malate-aspartate shuttle [184]. The malate-aspartate shuttle transfers reducing equivalents from the cytoplasm to the mitochondria and is also involved in the pathways for aspartate synthesis in cells (see Figure S2 in the Supplementary Materials). Our hypothesis is that the “*β*-oxidation shuttle” is expressed when the redox state of the matrix reaches the point of inhibition of *α*-KGDH and NAD-dependent IDH (or at least NAD-dependent IDH only), but MDH2 is not inhibited. A further increase in the NADH/NAD^+^ ratio may inhibit MDH2, which will stop simultaneously three processes: the malate-aspartate shuttle, the synthesis of aspartate, and the citrate-malate shuttle (see Figure S2 in the Supplementary Materials). Which of these three processes is most important for the survival of cancer cells? Surprisingly, this is not the provision of reducing equivalents for the ATP synthesis (not malate-aspartate shuttle or “*β*-oxidation shuttle”), but the synthesis of aspartate [174]. Cancer cells need many biochemical processes to grow and survive. Two of them are essential and are provided by mitochondria: (i) the synthesis of citrate, which is necessary for the synthesis of lipids (phospholipids, sphingolipids, coenzyme Q, and cholesterol) and (ii) the synthesis of aspartate, which is necessary for the synthesis of purines, pyrimidines, and ultimately DNA.

Partial defects in complexes I and III should exert pressure on the Q10H_2_/Q10 ratio and should increase the inhibitory effect of *β*-oxidation on SDH. Inhibition of ETC will also increase the NADH/NAD^+^ ratio to inhibit *α*-KGDH and NAD-dependent IDH. In fact, some mutations in the subunits of complex I that are responsible for its deficient activity lead to stimulation of tumor growth and make cancer cells highly metastatic [219]. However, further inhibition of ETC could cause problems even for cancer cells that are completely addicted to glycolysis and do not rely on mitochondria for ATP synthesis [219]. Although the “*β*-oxidation shuttle” can provide ATP to the cell, the main function of this metabolic pathway is to provide cytoplasmic citrate. Aspartate is crucial for the survival of cancer cells, as it is the main precursor for the synthesis of purines and DNA synthesis, respectively [174].

## 9. ATP Production and Oxygen Consumption by the “*β*-Oxidation Shuttle”

Assuming that “*β*-oxidation shuttle” is a key characteristic of some types of cancer cells, we should be able to compare its energy efficiency with that of glycolysis when combined with the combustion of pyruvate in the Krebs cycle. We calculated the energy efficiency and oxygen consumption of the “*β*-oxidation shuttle”, following mostly the accepted opinions about phosphate/oxygen (P/O) ratio from NADH+H^+^ and FADH_2_ (see Figure S1 in the Supplementary Materials) [220].

In these theoretical examples, we did not calculate the NADH produced by mitochondrial MDH2, because the cytoplasmic MDH1 consume NADH, and this calculation is not required for energy balancing (see Figure S3 in the Supplementary Materials). However, there should be a problem, as the number of mitochondrial NADH changes the result of the calculations, unless there is an existing mechanism for exporting reducing equivalents from mitochondria. In fact, there is such a mechanism associated with the “*β*-oxidation shuttle”. However, this is a mechanism for the transfer of reducing equivalents from NADH to NADP^+^ via NNT and subsequent export of reducing equivalents as NADPH by the citrate-*α*-KG shuttle (see Figure S3 in the Supplementary Materials).

If there really is a mechanism for transferring reducing equivalents from NADPH to NAD^+^ in the cytoplasm, then we have the right to exclude NADH produced by MDH2 from the calculations. We believe that this mechanism is the polyol pathway, which is a major characteristic of cancer cells (see Figure S3 in the Supplementary Materials). The reason for the existence of this pathway in cancer cells is still not well explained, except with the high-glucose availability in them [215–217].

The comparison between the “*β*-oxidation+Krebs cycle” with the “*β*-oxidation shuttle” shows that partial combustion of palmitate in the “*β*-oxidation shuttle” produces 26 moles of ATP, while its combustion in the “*β*-oxidation+Krebs cycle” produces 98 moles of ATP from 1 mole of palmitate (Table 1). Therefore, this is 3.76 times less ATP produced in the “*β*-oxidation shuttle” versus the “*β*-oxidation+Krebs cycle”. The comparison between the standard glucose combustion in the “glycolysis+Krebs cycle+malate-aspartate shuttle (MAS)” with the “*β*-oxidation shuttle” shows that the oxygen consumption per mole of ATP is 0.269 moles of oxygen in palmitate partial oxidation versus 0.1875 in glucose oxidation, which is approximately 1.43 times higher oxygen consumption in the “*β*-oxidation shuttle” (see Figure S1-A versus Figure S1(B) in the Supplementary Materials). The “*β*-oxidation shuttle” shows the lowest P/O ratio of 1.86 (Table 1).

However, this balance cannot be final, as the “*β*-oxidation shuttle” has a final product—acetyl-CoA. In order to calculate the actual energy efficiency and the actual oxygen consumption in a metabolic pathway that has a final product, the energy required to utilize the final product must also be included in the calculations. For example, in anaerobic glycolysis, we do not calculate the energy production until pyruvate production, but we should consider the conversion of pyruvate to lactate, which consumes two moles of NADH acquired in a previous step. In the case of the “*β*-oxidation shuttle”, acetyl-CoA can be utilized in synthetic processes and the energy cost of this synthesis should be included in the calculations. One of the easiest options for utilizing the final product acetyl-CoA is through the synthesis of fatty acids.

Theoretically, we can consider the coexistence of the two processes, “FAS+*β*-oxidation shuttle”, as a separate metabolic cycle when *β*-oxidation is overactivated, and the Krebs cycle is inhibited (see Figure S1(C) in the Supplementary Materials). For this artificial process, we can calculate that it is energetically possible and relies only on the availability of the NADPH. This NADPH could be produced in PPP or reductive glutaminolysis, if combined with NADPH-dependent isocitrate dehydrogenase and isocitrate-dependent NADPH exporting shuttle.

The ability that cancer cells can express both metabolic pathways simultaneously, FAS and mFAO, sounds irregular. We are accustomed to assuming that it is not energetically profitable to synthesize a substance and decompose it at the same time.

Typically, much more energy is used for synthesis than is released during the decomposition of the same compound. However, this rule does not apply when the energy comes from NADPH. This cycle is energetically beneficial in some extent since *β*-oxidation of palmitate produces more ATP compared to the amount of ATP spent on its synthesis (see Figure S1(C) in the Supplementary Materials). The energy difference is covered by NADPH, which can be produced in several ways. This artificial “*β*-oxidation shuttle+FAS cycle” could be considered as a process for conversion NADPH energy into energy of some amount of ATP.

In this article we do not discuss whether this cycle really exists or in how many types of cancer cells it appears. Our goal is to use it as a model for calculating energy efficiency and oxygen consumption in irregularly activated mitochondrial *β*-oxidation, when the Krebs cycle is inhibited, and the final product acetyl-CoA is used in the synthesis of fatty acids.

In the cycle “FAS+*β*-oxidation shuttle”, when NADPH is coming only from PPP, the oxygen consumption is 0.64 moles per mole of ATP (Table 1). “FAS+*β*-oxidation shuttle” consumes about 3.4 times more oxygen per mole of ATP than “glycolysis+Krebs cycle+MAS”.

Whether “*β*-oxidation shuttle+FAS” cycle really exists in cancer cells?

We are not claiming that the “*β*-oxidation shuttle+FAS” is a real cycle. Probably, there are additional reasons why this cycle could not occur as shown in the Figure S1-B (see the Supplementary Materials section). For example, the lipotoxicity of palmitate and stearate may be an important reason. Maybe that is why some cancer cells are addicted to extrinsic fats. However, this does not prevent the coexistence of the two metabolic pathways FAS and “*β*-oxidation shuttle” and does not significantly change the above calculations. Acetyl-CoA produced in the “*β*-oxidation shuttle” must be consumed and the cost should be paid. This cost is the energy consumed for the utilization of acetyl-CoA and this could be the energy for FAS. In one study, berberines were found to be inhibitors of fatty acid binding proteins—a family of proteins that transport fatty acids across the membranes [221]. Given this fact, cancer cells appear to be more dependent on external fatty acids than on their own.

## 10. Additional Factors That Could Interfere with the “*β*-Oxidation Shuttle” and Glutaminolysis

### 10.1. Deffects in the Krebs Cycle Enzymes and Mutations in ETC

As already discussed, *β*-oxidation alone may not have enough power to express the mFAO outside the Krebs cycle, and may not have enough force to increase the redox state of the mitochondrial matrix, in the absence of additional defects in the metabolic pathways and/or ETC. In accordance with this, many dysregulations of SDH, FH, and *α*-KGDH are described in cancer [102, 222–224].

The ETC defects in cancer cells are widely discussed and there are many publications on the subject. One of the latest review articles on this topic dates from 2020 [225]. As already mentioned, inhibition of ETC will increase the NADH/NAD^+^ ratio to inhibit *α*-KGDH and NAD-dependent IDH. In fact, some mutations in the subunits of complex I lead to stimulation of tumor growth and make cancer cells highly metastatic [219]. However, further inhibition of ETC could cause problems even for cancer cells that are completely addicted to glycolysis and do not need mitochondria as a primary source for ATP synthesis but need aspartate for proliferation [219].

Below, we would like to highlight some points that we consider important, although they are less discussed in the literature.

### 10.2. Inhibition of ETC and Glutaminolysis

A comprehensive analysis of the oxidative and reductive pathways of glutaminolysis, depending on the dysfunctions in SDH, fumarase, and mutations in complex III, was published by Mullen et al. [226]. The authors found experimentally that all mutated strains express reductive carboxylation of *α*-KG. Their study clearly demonstrates that the reductive carboxylation of *α*-KG requires the production of NADH and NNT. It has been found that the NADH required for the reductive carboxylation is derived from the oxidative reaction of *α*-KGDH, and the accumulated succinate comes from glutamate. The authors concluded that the oxidative metabolism of *α*-KG is required for reductive carboxylation [226]. They also found that cells with complex III mutation display predominantly reductive carboxylation and oxaloacetate comes from increased pyruvate carboxylation by pyruvate carboxylase. In contrast, oxidative pathways of glutaminolysis dominate in wild-type cells [226]. What is the difference between wild type strains and complex III mutated strains? In our opinion, it should be the inhibition of SDH by the reductive state of the Q-pool, which can also increase the reductive pressure through complex I and increased NADH levels.

Thus, in the study of Mullen et al. described above, the production of NADH by *α*-KGDH seems sufficient to express the reductive carboxylation. However, the possible contribution of *β*-oxidation should be investigated. Since the driving force of the reductive carboxylation is highly reduced state of NAD(P)H pools, at this state, *α*-KGDH should be at least partially inhibited. Moreover, in hypoxia and hypoglycemia, the power of *α*-KGDH to produce NADH seems insufficient. The experiments were performed with cells cultured with limited ability to use fatty acids in their metabolism. In solid tumors, the environment of cancer cells is predominantly hypoxic and hypoglycemic [104], and fatty acids could be highly available. In hypoglycemic conditions, *β*-oxidation is most likely to be activated. In hypoxic conditions, ETC is much more inhibited and NADH levels could reach the level of *α*-KGDH inhibition. In this case, only *β*-oxidation can supply the mitochondria with the necessary NADH for the reductive carboxylation of *α*-KG.

On the other hand, the glutaminolysis and *β*-oxidation seem equally overactivated when mitochondrial catabolism of pyruvate is substantially decreased. Veliova et al. discovered that pharmacological inhibition of mitochondrial pyruvate carrier (MPC) in brown adipocytes was sufficient to increase ATP synthesis fueled by mFAO and ATP consumption by FAS [227]. Furthermore, they found that glutamine consumption and the malate-aspartate shuttle were necessary to increase the energy expenditure caused by MPC inhibition. This suggests that oxidative glutaminolysis is required for the operation of the “*β*-oxidation shuttle” or vice versa; oxidative glutaminolysis needs expression of the “*β*-oxidation shuttle” to provide sufficient acetyl-CoA to the Krebs cycle. Vacanti et al. also observed in other cells that the knockdown or inhibition of MPC was sufficient to increase both glutaminolysis and mFAO and to increase the substrate for de novo lipogenesis [228]. Both processes (glutaminolysis and *β*-oxidation) appear to increase due to the lack of pyruvate degradation products—oxaloacetate and acetyl-CoA. MPC inhibition increases oxidative glutaminolysis, which directly supports the production of oxaloacetate and indirectly, the production of acetyl-CoA by malic enzyme [228]. However, the supply of acetyl-CoA produced only by malic enzyme could be insufficient, because MPC inhibition also increases *β*-oxidation flux in the Krebs cycle [228]. The production of acetyl-CoA by *β*-oxidation should be even more necessary for cancer cells and in hypoxia, when PDH is substantially inhibited.

**Figure 13:**
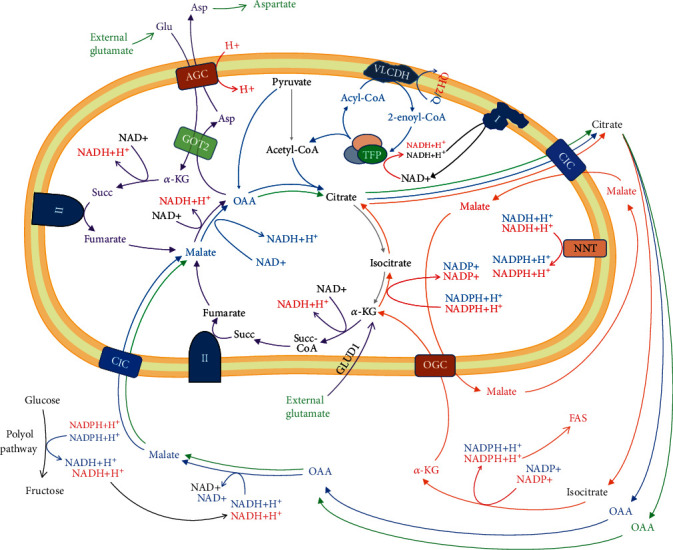
Beta-oxidation shuttle and glutaminolysis pathways. The blue arrows indicate *β*-oxidation shuttle. The purple arrows indicate oxidative glutaminolysis and aspartate synthesis pathways. The green arrows indicate anaplerosis of “*β*-oxidation shuttle” by oxidative and reductive glutaminolysis. The orange arrows indicate citrate-isocitrate shuttle. AGC: aspartate-glutamate carrier; Asp: aspartate; CIC: mitochondrial citrate carrier; FAS: fatty acid synthesis; Glu: glutamate; GLUD1: glutamate dehydrogenase 1; GOT2: glutamic-oxaloacetic transaminase 2; a-KG: a-ketoglutarate; NNT: NAD(P) transhydrogenase; OAA: oxaloacetate; OGC: oxoglutarate carrier; Succ: succinate; TFP: trifunctional protein; VLCDH: very long-chain dehydrogenase.

Another interesting point in the study of Mullen et al. is that wild-type cancer cells without defects in ETC, SDH, or FH use mainly oxidative glutaminolysis [226]. Thus, it seems to have cancer cells with functional Krebs cycle and functional ETC, but with activated glutaminolysis. What is the reason for glutaminolysis in this situation? It is difficult to assume that the cause of activated glutaminolysis can be in the inhibited PDH, which is the most discussed impairment as the cause of the Warburg effect. Inhibition of PDH is thought to be a major reason of increased glycolysis, but within the mitochondrial matrix, it can only limit the availability of acetyl-CoA. Rather, oxaloacetate deficiency may activate glutaminolysis (Figure 13). Oxaloacetate deficiency may result from increased aspartate synthesis. However, oxidative glutaminolysis cannot replace the production of acetyl-CoA in cancer cells because they need citrate production for the activated FAS and mevalonate pathway. We propose that the redox state of the mitochondrial matrix could have different degrees. Glutaminolysis comes mainly from first-grade impairment of the redox state of the matrix and NAD-dependent IDH is the most sensitive enzyme. This could be the point of inhibition of the Krebs cycle, which expresses glutaminolysis to restore the cycle from the point where it was inhibited. Moreover, the lack of acetyl-CoA could be covered only by *β*-oxidation when PDH is inhibited. As already stated, expression of glutaminolysis may be inseparable from the “*β*-oxidation shuttle” when pyruvate availability is limited. Restrictions in pyruvate catabolism may increase both glutaminolysis and *β*-oxidation [228]. At the same time, the substrates for FAS increase [228]. PDH deficiency further increases the dependence of cells on fatty acid catabolism, and PDH is not required for cell proliferation, as described by Rajagopalan et al. [229]. This suggests that inhibition of NAD-dependent IDH may be caused by increased *β*-oxidation. At this point, oxidative glutaminolysis could restore the normal function of the Krebs cycle together with increased cataplerosis of citrate. Further, when SDH or FH is inhibited or ETC is overloaded with reducing equivalents such as Q10H_2_, the reductive carboxylation of *α*-KG is expressed, as described by Mullen et al. [226]. At this point, oxidative glutaminolysis could still produce NADH via *α*-KGDH with the coexistence of a reductive pathway, but again together with the “*β*-oxidation shuttle”. The dependence on the “*β*-oxidation shuttle” should increase with increased ETC dysfunctions and increased NADH levels due to the increased degree of *α*-KGDH inhibition. The second level of reduced state of the mitochondrial matrix should inhibit *α*-KGDH and express reductive carboxylation depending only on the “*β*-oxidation shuttle”.

Another evidence that the expression of glutaminolysis may be inseparable from the “*β*-oxidation shuttle” comes from Luis et al. [53]. As already noted, the adipocyte-supplemented medium significantly increases the viability and proliferation of breast cancer cells (MCF-7), as well as their migration and aggressiveness. Cancer cells in a high-fat environment, contrary to normal expectations, increase lactate production and glutamine consumption when mFAO is overactivated. We would like to emphasize the word “overactivated” because our opinion is that “mitochondrial *β*-oxidation over the energy needs of the cell” is the driving force behind all these peculiarities of cancer metabolism. Evidence of this is the ability to stimulate reductive glutaminolysis, as well as the consumption of fatty acids not only in cancer cells but also in normal cells when pyruvate is not available and *α*-KGDH is inhibited. Recently, the DeBerardinis's group investigated metabolic disorders due to defects in the gene encoding lipoyltransferase-1 (LIPT1), which catalyzes the transfer of lipoic acid to the E2 subunits of 2-keto dehydrogenases, PDH and *α*-KGDH [230]. Fibroblasts with LIPT1 mutations have impaired pyruvate metabolism and impaired Krebs cycle in the *α*-KGDH segment. The metabolic consequence of this mutation is the increased need of pathways such as mFAO and glutaminolysis. When the substrates (fatty acids and glutamine) are available, fibroblasts are viable and proliferate. The glutaminolysis pathway is predominantly reductive. The contribution of glutamine to FAS is also increased in LIPT1-deficient cells, but fatty acid synthesis in these cells is overall decreased, compared to LIPT1-expressing cells [230]. In general, the increased coexistence of mFAO with decreased FAS is evidence of normal function of the AMPK-ACC2 mechanism. This mechanism prevents the cell from entering a futile “mFAO+FAS” cycle. mFAO is limited to the need of cell for ATP, and FAS is limited in LIPT1-deficient fibroblasts. Without persistent overactivated *β*-oxidation, the reduced state of the mitochondrial matrix is difficult to achieve. It should be also noted that in this case, we are talking about fibroblasts that are not cancer cells.

We can learn a lot about reductive carboxylation from studies on aspartate synthesis. The synthesis of aspartate in cancer cells is very important for their survival [174]. There are three pathways for the synthesis of aspartate from glutamate. Two of them are oxidative. Most cancer cells use glutamate dehydrogenase 1 (GLUD1) to convert glutamine-derived glutamate into *α*-KG in the mitochondria to supply the Krebs cycle [185] (Figure 13). However, Son et al. reported that pancreatic cancer cells use predominantly the GOT2-dependent pathway to convert glutamate to *α*-KG [185] (Figure 13). GOT2-derived aspartate is transported into the cytoplasm where it can be converted into oxaloacetate by aspartate transaminase (GOT1). The oxaloacetate is converted into malate and then malate into pyruvate by malic enzyme 1 (ME1), to increase the NADPH/NADP^+^ ratio. This redox ratio has been found to be important because the knockdown of cytoplasmic ME1 significantly inhibits not only the production of NADPH but also the formation of colonies of pancreatic cancer cells. The authors also found that knockdown of GOT1 and ME1 markedly decreased the cellular NADPH/NADP^+^ ratio, while inhibition of other cytosolic sources of NADPH (G6PD or isocitrate dehydrogenase, IDH1) had no effect on the NADPH/NADP^+^ ratio.

These observations indicate that the reductive carboxylation of *α*-KG is not dominant in pancreatic cancer cells. Instead, the oxidative synthesis of aspartate and associated NADPH production is most important in these cells. However, Zarei et al. observed that under stress conditions such as glucose deprivation, the factor that regulates acute survival processes, human antigen R (HuR) (encoded by the ELAVL1 gene) increases antioxidant defense through post-transcriptional stabilization of the NADPH-generating enzyme IDH1 in pancreatic cells [231, 232]. Moreover, overexpression of IDH1 in HuR-deficient cells was sufficient to fully restore chemoresistance under low nutrient amount. These facts suggest that some enzymes, such as IDH1, can be limiting factors for the expression of the citrate-*α*-KG shuttle. The shuttle could be limited by the availability of IDH1. In this case, the increased glutaminolysis could increase mainly the oxidative flux in the Krebs cycle instead of the reductive flux. However, under stress conditions, the citrate-*α*-KG shuttle could play an important role. In addition, mitochondrial *β*-oxidation is likely to be more activated under stress conditions along with IDH1, especially in glucose deprivation.

Zarei et al. found that GOT1 knockdown resulted in increased glutamine-derived aspartate in pancreatic cancer cells [231]. The same is observed by Birsoy et al. earlier in leukemia cells (Jurkat) [233]. In this study, GOT1-deficient Jurkat cells produced a significantly large amount of aspartate predominantly by the oxidative pathway, which was found to be dependent on GOT1 and GOT2. It is interesting why GOT1 deficiency is involved in increased aspartate synthesis, as GOT1 is not part of the GOT2-dependent pathway of aspartate synthesis (Figure 13). However, GOT1 is an important part of the malate-aspartate shuttle (Figure 13) (see Figure S2 in the Supplementary Materials). The driving force of this shuttle is the aspartate/glutamate carrier, which is dependent on proton gradient and works only in one direction [184, 234]. In the absence of GOT1, the malate-aspartate shuttle is not functional, but aspartate/glutamate carrier continues to operate as part of the aspartate synthesis pathway. The part of the aspartate/glutamate carrier that is engaged to work for malate-aspartate shuttle begins to work only for aspartate synthesis (Figure 13).

Finally, the redox state of the mitochondrial matrix may reach the point where MDH2 is inhibited. In the study of Birsoy et al., mitochondrial synthesis of aspartate by GOT2 can be completely abrogated by phenformin and inhibition of ETC kills GOT1-deficient Jurkat cells [233]. However, in the study of Sullivan et al., *α*-ketobutirate, which is a mitochondrial electron acceptor and produces NAD^+^ in the matrix, saves cancer cells with inhibited ETC [174]. These two studies indicate that the redox state of the mitochondrial matrix of cancer cells could be easily overcharged by reducing equivalents to inhibit aspartate synthesis. Oxidative synthesis depends on the work of mitochondrial GOT2 and the presence of mitochondrial oxaloacetate. Inhibition of the forward reduction of malate to oxaloacetate catalyzed by MDH2 should be the only reason for the inhibited oxidative synthesis of aspartate due to inhibition of complex I. Thus, many inhibitors of complex I are being studied as anticancer drugs. For example, phenformin used in the study of Birsoy et al. [233] is a complex I inhibitor and was used as antidiabetic drug for 20 years until 1977. It is not as toxic to noncancer cells, but phenformin has been withdrawn from the market because it can cause lactic acidosis in some diabetic patients. These observations support the conclusion that inhibition of complex I may cause a Warburg effect in noncancer cells, but in cancer cells that are overcharged with reducing equivalents, it may cause a suppression of proliferation through decreased availability of aspartate.

## 11. Concluding Remarks

In conclusion, the findings described and analyzed in this article indicate that irregular overactivation of mitochondrial *β*-oxidation may switch to the “*β*-oxidation shuttle” due to insufficient activity of key enzymes of the Krebs cycle or PDH or ETC complexes. On the other hand, overactivation of mitochondrial *β*-oxidation can cause dysfunctions in the ETC, as well as in the Krebs cycle, especially in hypoxic conditions. The resulting overreduced redox state of the cells triggers compensatory pathways for nonmitochondrial ATP production and utilization of NADH, which is anaerobic glycolysis.

The subsequent return to normal combustion of pyruvate and fatty acids may not occur easily and the delay may cause diseases, including carcinogenesis. It is still unclear what is the key event that turns cells into malignancy and makes this metabolic and redox state irreversible. One clue for the key events in transformation of cells to cancerous seems to be the increased utilization of overproduced citrate by the FAS and mevalonate pathway. Nevertheless, targeting and modulating the altered mitochondrial redox state by redox-active substances seems to be valuable therapeutic alternative, especially in cancer [235].

Surprisingly, the “*β*-oxidation shuttle” fits well with the Warburg effect, which has not yet been convincingly explained. When cancer cells are removed from their natural hypoxic environment, they could have normal oxygen consumption, but still prefer to convert glucose anaerobically. Increased oxygen demand of the “*β*-oxidation shuttle”, combined with the inhibited activity of PDH and partially inhibited *β*-oxidation enzymes, can lead to seemingly normal oxygen consumption combined with lactate production, inefficient mitochondrial ATP synthesis, and huge cataplerosis. This explains well the Warburg effect, as well as the uncontrolled growth and proliferation. Glycolysis and OXPHOS should not be antagonized, because OXPHOS can coexist together with anaerobic glycolysis when the “*β*-oxidation shuttle” is expressed. The possible inefficient synthesis of ATP in the “*β*-oxidation shuttle” provides conditions for some cancer cells to be dependent simultaneously on OXPHOS and glycolysis. As much inefficient is the mitochondrial ATP synthesis in relation to the “*β*-oxidation shuttle”, as more cancer cells should be dependent on glycolysis. The main role of the “*β*-oxidation shuttle” should be the export of citrate and the huge cataplerosis—the main characteristic of cancer cells. On the other hand, we consider that the overreduced mitochondrial matrix is a consequence of overactivated mitochondrial *β*-oxidation, and at the same time, a major cause of the expression of *β*-oxidation outside the Krebs cycle. Lactate dehydrogenase helps cancer cells to decrease the reduced state of the mitochondrial matrix by producing NAD^+^. This explains anaerobic glycolysis as a compensatory mechanism not only for the ATP production but also for mitigation the consequences of the enormously increased redox state of the mitochondrial matrix.

The “*β*-oxidation shuttle” could be expressed to some extent under control in noncancerous proliferating cells, as well as in certain types of immune cells and embryonic cells. However, this expression should be reversible, while in cancer cells it should be irreversible. The “*β*-oxidation shuttle” may be tightly connected with some chronic diseases such as diabetes 2, obesity, fatty liver disease, and cardiac hypertrophy, but it is difficult to predict whether this "weird" metabolic pathway is reversible in these diseases. All these assumptions need experimental validation. It needs to be clarified whether it is possible to restore normal functionality of mitochondria after they fall in this metabolic dysfunction.

The role of the “*β*-oxidation shuttle” in impaired cancer metabolism should be an object of future studies. This could be a crucial metabolic “secret” of cancer.

## Figures and Tables

**Table 1:** P/O ratio and oxygen consumption in the combustion of glucose and palmitate.

Metabolic pathway	P/O ratio	Oxygen consumption per mol ATP
Glycolysis+Krebs cycle+MAS	2.66	0.1875 moles O_2_
*β*-oxidation+Krebs cycle	2.13	0.235 moles O_2_^∗^
*β*-oxidation shuttle	1.86	0.269 moles O_2_^∗∗^
*β*-oxidation shuttle+FAS	0.79	0.640 moles O_2_^∗∗∗^

P/O: phosphate/oxygen ratio; MAS: malate-aspartate shuttle; FAS: fatty acid synthesis. ^∗^1.25 times higher O_2_ consumption compared to “glycolysis+Krebs cycle+MAS”. ^∗∗^1.43 times higher O_2_ consumption compared to “glycolysis+Krebs cycle+MAS”. ^∗∗∗^3.41 times higher O_2_ consumption compared to “glycolysis+Krebs cycle+MAS”.

## Data Availability

Data in the supporting information are available on request via the following e-mails: bakalova.rumiana@qst.go.jp (Dr. Rumiana Bakalova) or zh_zhelev@yahoo.com (Dr. Zhivko Zhelev).

## Abbreviations

ACAD: Acyl-CoA dehydrogenase
ACAT1: Acetyl-CoA acetyltransferase
ACC1 and ACC2: Acetyl-CoA carboxylases 1 and 2
ACLY: ATP-dependent citrate lyase
AMPK: Adenosine monophosphate-activated protein kinase
CIC: Citrate/isocitrate carrier
CPT1 and CPT2: Carnitine palmitoyl transferases 1 and 2
ETC: Electron transport chain
mFAO: Mitochondrial fatty acid oxidation
FAS: Fatty acid synthesis
FH: Fumarate hydratase
HIF-1*α*: Hypoxia-inducible factor one alpha
GAPDH: Glyceraldehyde 3-phosphate dehydrogenase
*α*-KG: Alpha-ketoglutarate
*α*-KGDH: Alpha-ketoglutarate dehydrogenase
LCAD: Long-chain acyl-CoA dehydrogenase
MCD: Malonyl-CoA decarboxylase
MDH1 and MDH2: Malate dehydrogenases 1 and 2
MPC: Mitochondrial pyruvate carrier
NNT: Nicotinamide nucleotide transhydrogenase
OXPHOS: Oxidative phosphorylation
PC: Pyruvate carboxylase
PDH: Pyruvate dehydrogenase
PDK: Pyruvate dehydrogenase kinase
PDP: Pyruvate dehydrogenase phosphatase
PPP: Pentose phosphate pathway
Q and QH2: Coenzyme Q10 (oxidized and reduced forms)
RET: Reverse electron transport
ROS: Reactive oxygen species
SDH: Succinate dehydrogenase
SIRT: Sirtuin
TFP: Mitochondrial trifunctional fatty acid oxidation enzyme
VLCAD: Very long-chain acyl-CoA dehydrogenase.

## Supplementary Materials

Supplementary Materials Supporting information contains Figures S1, S2, and S3. Figure S1 illustrates energy balance and oxygen consumption of several metabolic pathways: (A) “glycolysis+Krebs cycle+MAS”, (B) “*β*-oxidation shuttle”, and (C) “FAS+*β*-oxidation shuttle”. Figure S2 illustrates the relationship between the *β*-oxidation shuttle, malate-aspartate shuttle, and glutaminolysis pathways. Figure S3 illustrates how polyol pathway solves the redox equation of the *β*-oxidation shuttle.
