# Supplementary material for: A “Weird” Mitochondrial Fatty Acid Oxidation as a Metabolic “Secret” of Cancer
Source: Oxid Med Cell Longev. 2022 Feb 8;2022:2339584. doi: 10.1155/2022/2339584 (PMC8847026; doi:10.1155/2022/2339584)
Supplement: Supplementary Materials — Supporting information contains Figures S1, S2, and S3. Figure S1 illustrates energy balance and oxygen consumption of several metabolic pathways: (A) “glycolysis+Krebs cycle+MAS”, (B) “β-oxidation shuttle”, and (C) “FAS+β-oxidation shuttle”. Figure S2 illustrates the relationship between the β-oxidation shuttle, malate-aspartate shuttle, and glutaminolysis pathways. Figure S3 illustrates how polyol pathway solves the redox equation of the β-oxidation shuttle. [file 2339584.f1.docx]

**Supporting Information**

**A “weird” mitochondrial fatty acid oxidation as a metabolic “secret” of cancer**

Zhivko Zhelev^1,2,3^, Ichio Aoki^1^, Dessislava Lazarova^4^, Tatyana Vlaykova^2^, Tatsuya Higashi^1^, Rumiana Bakalova^1^*

^1^Department of Molecular Imaging and Theranostics, National Institutes for Quantum Science and Technology (QST), Chiba 263-8555, Japan; ^2^Faculty of Medicine, Trakia University, Stara Zagora 6000, Bulgaria; ^3^Institute of Biophysics and Biomedical Engineering, Bulgarian Academy of Sciences, Sofia 1113, Bulgaria; ^4^Faculty of Medicine, Sofia University “St. Kliment Ohridski”, Sofia 1407, Bulgaria.

**Figure S1.** **(A)** Energy balance and oxygen consumption of “glycolysis + Krebs cycle + MAS”. **(B)** Energy balance and oxygen consumption of β-oxidation shuttle. **(C)** Energy balance of an artificial cycle of “FAS + β-oxidation shuttle” when NADPH is coming from pentose phosphate pathway (PPP).

**(S1-A) Energy balance and oxygen consumption of “glycolysis + Krebs cycle + MAS”**


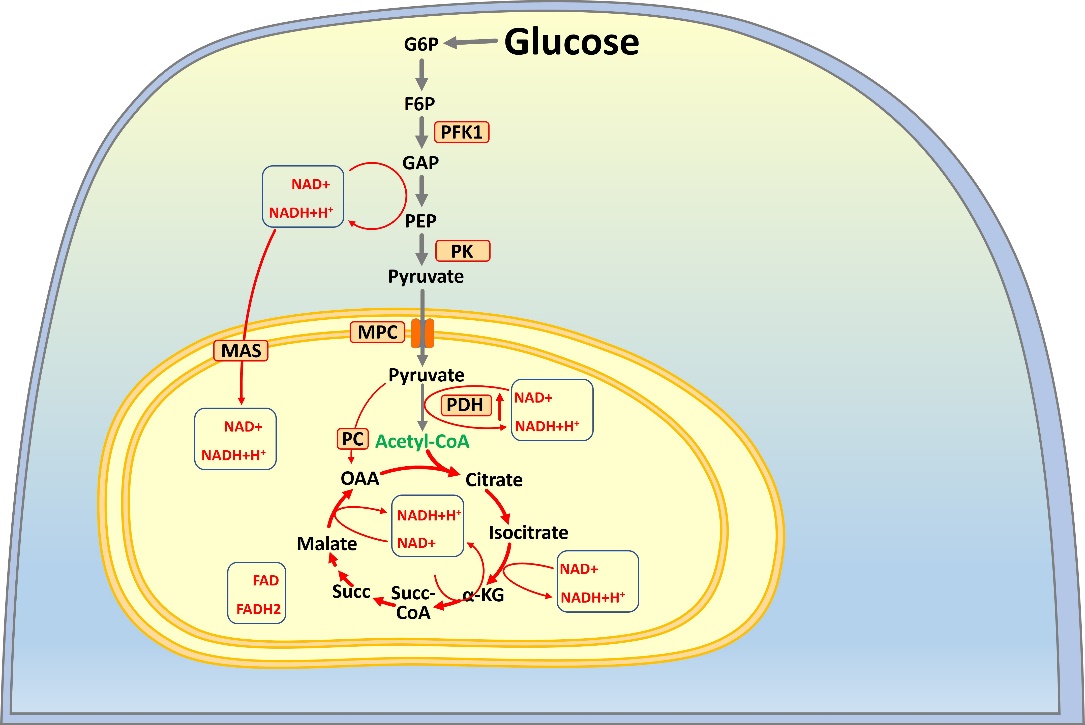


*Legend: F6P – fructose-6-phosphate/fructose-1,6-bisphosphate; G6P – glucose-6-phosphate; GAP – glyceraldehyde-3-phosphate; a-KG – a-ketoglutarate; MPC – mitochondrial pyruvate carrier; MAS – malate-aspartate shuttle; OAA – oxaloacetate; PDH – pyruvate dehydrogenase; PC – pyruvate carboxylase; PFK1 – phosphofructokinase-1; PK – pyruvate kinase; PEP – phosphoenolpyruvate; Succ – succinate*

**Calculations:**

**Glucose oxidation by “glycolysis + Krebs cycle + MAS”** can be represented by the following equations:

1. From Glucose to Pyruvate:

**Glucose + 2NAD^+^ + 2ADP + 2Pi → 2Pyruvate + 2NADH+H^+^ + 2ATP**

1. From Pyruvate to Acetyl-CoA by PDH:

**2Pyruvate** + 2**SH-CoA** +**2NAD+** → **2Acetyl CoA** **+** **2NADH+H^+^ + 2CO_2_**

1. From Acetyl-CoA to NADH+H^+^ and FADH_2_ in the Krebs cycle:

**2Acetyl-CoA** + **6NAD+ + 2FAD + 2ADP + 2Pi** → **2CoA** + **4CO_2_** + **6NADH+H^+^ +**

**+ 2FADH_2_ +** **2ATP**

1. From NADH to ATP by ETC:

**10NADH+H^+^** **+ 5O_2_** +**25Pi** + **25ADP** → **10NAD^+^ +** **25ATP** **+** **10H_2_O**

**2FADH_2_** **+ O_2_** +**3Pi** + **3ADP** → **2FAD +** **3ATP** **+** **2H_2_O**

***Note:*** *All the above calculations were performed considering that: (i)****one molecule of NADH yields 2.5 molecules of ATP*** *and****one molecule of FADH_2_ yields 1.5 molecules of ATP****in the Electron Transport System; (ii) 1/2 O_2_ is required to oxidize one molecule of each of the two substances (NADH or FADH_2_) (as described by Hinkle^220^).*

**TOTAL BALANCE:**

**TOTAL amount of ATP produced in “glycolysis + Krebs cycle + MAS”: 32 moles ATP**

**TOTAL amount of oxygen consumed: 6 moles O_2_**

**P/O ratio: 2.66**

**Oxygen consumption per mol ATP: 0.1875 moles O_2_ per 1 mol ATP**

**(S1-B) Energy balance and oxygen consumption of β-oxidation shuttle**


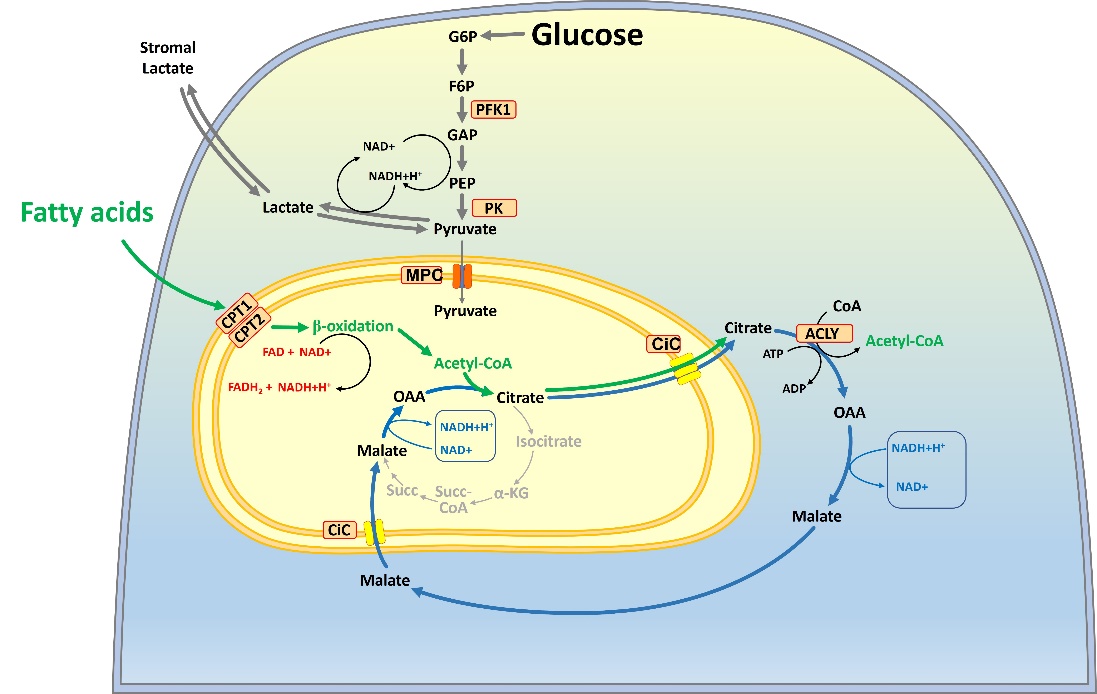


*Legend: ACLY – ATP citrate lyase; CIC – mitochondrial citrate carrier; CTP1 and CTP2 – carnitine palmitoyl transferases 1 and 2; F6P – fructose-6-phosphate/fructose-1,6-bisphosphate; G6P – glucose-6-phosphate; GAP – glyceraldehyde-3-phosphate; a-KG – a-ketoglutarate; MPC – mitochondrial pyruvate carrier; OAA – oxaloacetate; PFK1 – phosphofructokinase-1; PK – pyruvate kinase; PEP – phosphoenolpyruvate; Succ – succinate.*

**Calculations:**

1. **Partial oxidation of Palmitoyl-CoA** can be represented by the following equations:

**Palmitoyl CoA** + **7CoA-SH** + **7FAD** + **7NAD^+^**+ **7H_2_O** **→ 8Acetyl-CoA** **+**

**+ 7FADH_2_** **+** **7NADH+H^+^**

1. Converting NADH and FADH_2_ to their corresponding ATP equivalents:

**7NADH+H^+^** + **3.5O_2_** +**17.5Pi** + **17.5ADP** → **7NAD^+^** + **17.5ATP** + **7H_2_O**

**7FADH_2_** + **3.5O_2_** +**10.5Pi** + **10.5ADP** → **7FAD** + **10.5ATP** + **7H_2_O**

**or**

**7NADH+H^+^** + **7FADH_2_** + **7O_2_** +**28Pi** + **28ADP** → **7NAD+** + **7FAD** +

+ **28ATP** + **14H_2_O**

***Note: Activation of Palmitoyl-CoA****consumes 2 high energy bonds because ATP is used and (AMP + PP) is produced.*

**TOTAL BALANCE:**

**TOTAL amount of Acetyl-CoA and ATP produced in the β-oxidation shuttle: 8 moles Acetyl-CoA + 26 moles of ATP**

**TOTAL amount of oxygen consumed: 7 moles O_2_**

**P/O ratio: 1.86**

**Oxygen consumption per mol ATP: 0.269 moles O_2_ per 1 mol ATP**

***Note: β-oxidation shuttle versus “glycolysis + Krebs cycle + MAS:*** ***1.43 times more O_2_ consumed in the β-oxidation shuttle compared to “glycolysis + Krebs cycle + MAS”***

**(S1-C) Energy balance of an artificial cycle of “FAS + β-oxidation shuttle” when NADPH is coming from PPP**


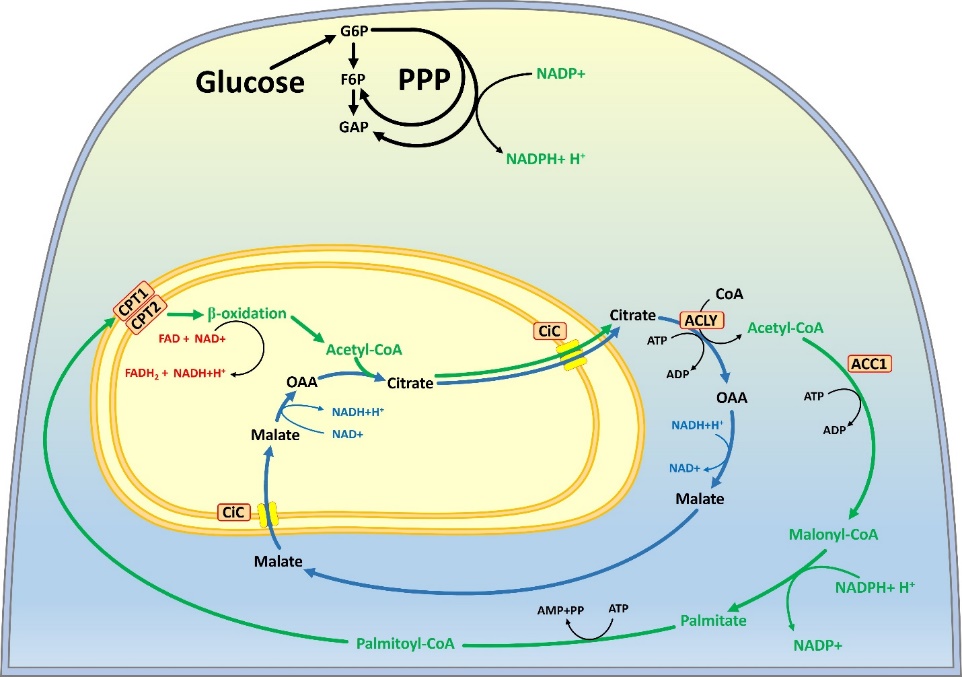


*Legend: ACC1 – acetyl-CoA carboxylase 1; ACLY – ATP citrate lyase; CIC – mitochondrial citrate carrier; CTP1 and CTP2 – carnitine palmitoyl transferases 1 and 2; F6P – fructose-6-phosphate/fructose-1,6-bisphosphate; G6P – glucose-6-phosphate; GAP – glyceraldehyde-3-phosphate; OAA – oxaloacetate; PPP – pentose phosphate pathway.*

**Calculations:**

1. **In β-oxidation:**

Oxidation of Palmitic acid:

**TOTAL amount of Acetyl-CoA and ATP produced: 8 moles Acetyl CoA** + **26 moles ATP**

**TOTAL amount of oxygen consumed: 7 moles O_2_**

1. **In fatty acid synthesis:**

Synthesis of Palmitic acid:

1. For conversion of 8 Citrate moles to **8 Acetyl-CoA** + Oxaloacetate: **8 ATP** are required
2. For activation of Acetyl-CoA to Malonyl-CoA: **7 ATP** are required
3. At each turnover 2 NADPH+H^+^ = **14 NADPH+H^+^** are required

**TOTAL needs: 8 moles Acetyl CoA, 15 moles ATP,** and **14 moles NADPH+H^+^**

1. **PPP & FAS+mFAO cycle:**

**TOTAL amount of ATP produced in FAS+mFAO cycle: 11 moles ATP**

**TOTAL amount of oxygen consumed: 7 moles O_2_**

**TOTAL amount of glucose consumed: 1.16 moles**

**P/O ratio: 0.79**

**Oxygen consumption per mol ATP: 0.64 moles O_2_ per 1 mol ATP**

***Note: PPP & FAS+mFAO cycle versus “glycolysis + Krebs cycle + MAS: 3.41 times more O_2_ consumed in the PPP & FAS+mFAO cycle compared to “glycolysis + Krebs cycle + MAS”***

*******

**Energy balance and oxygen consumption of “β-oxidation + Krebs cycle + MAS”**

1. **Partial oxidation of Palmitoyl CoA** can be represented as following equations:

**Palmitoyl-CoA** + **7CoA-SH** + **7FAD** + **7NAD^+^**+ **7H_2_O** → **8Acetyl-CoA** +

+ **7FADH_2_** + **7NADH+H^+^**

1. Converting NADH and FADH_2_ to their corresponding ATP equivalents:

**7NADH+H^+^** + **3.5O_2_** +**17.5Pi** + **17.5ADP** → **7NAD+** + **17.5ATP** + **7H_2_O**

**7FADH_2_** + **3.5O_2_** +**10.5Pi** + **10.5ADP** → **7FAD** + **10.5ATP** + **7H_2_O**

**or**

**7NADH+H^+^** + **7FADH_2_** + **7O_2_** +**28Pi** + **28ADP** → **7NAD+** + **7FAD** + **28ATP** + **14H_2_O**

1. Converting 8Acetyl-CoA to NADH+H^+^ and FADH_2_ in the Krebs cycle:

**8Acetyl-CoA** +**24NAD+ + 8FAD + 8ADP + 8Pi** → **8CoA** + **16CO_2_** + **24NADH+H^+^ +**

**+ 8FAH_2_ + 8ATP**

1. Converting NADH to ATP by ETC:

**24NADH+H^+^** **+ 12O_2_** +**60Pi** + **60ADP** → **24NAD^+^ +** **60ATP** **+** **24H_2_O**

**8FADH2** **+ 4O_2_** +**12Pi** + **12ADP** → **8FAD +** **12ATP** **+** **8H_2_O**

***Note: Activation of Palmitoyl-CoA****consumes 2 high energy bonds because ATP is used and (AMP + PP) is produced.*

**TOTAL BALANCE:**

**TOTAL amount of ATP produced in the “β-oxidation + Krebs cycle + MAS”: 98 moles ATP**

**TOTAL amount of oxygen consumed: 23 moles O_2_**

**P/O ratio: 2.13**

**Oxygen consumption per mol ATP: 0.235 moles O_2_ per 1 mol ATP**


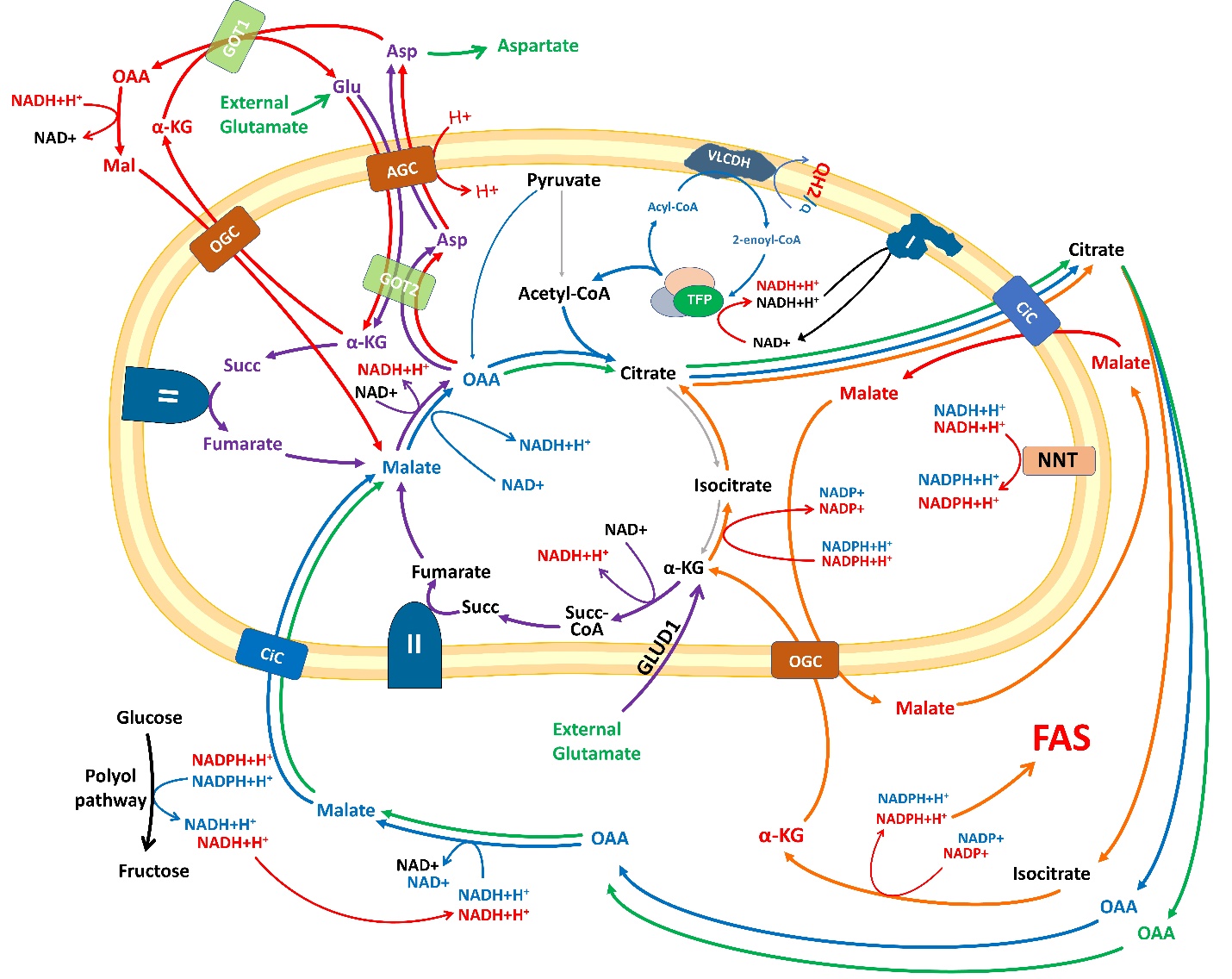


**Figure S2.** Beta-oxidation shuttle, malate-aspartate shuttle and glutaminolysis pathways. The blue arrows indicate β-oxidation shuttle. The purple arrows indicate oxidative glutaminolysis and aspartate synthesis pathways. The green arrows indicate anaplerosis of β-oxidation shuttle by oxidative and reductive glutaminolysis. The red arrows indicate malate-aspartate shuttle. The orange arrows indicate citrate-isocitrate shuttle. *Legend: AGC – aspartate-glutamate carrier; Asp – aspartate; CIC – mitochondrial citrate carrier; FAS – fatty acid synthesis; Glu – glutamate; GLUD1 – glutamate dehydrogenase 1; GOT2 – glutamic-oxaloacetic transaminase 2; a-KG – a-ketoglutarate; NNT – NAD(P) transhydrogenase; OAA – oxaloacetate; OGC – oxoglutarate carrier; Succ – succinate; TFP – trifunctional protein; VLCDH – very long chain acyl-CoA dehydrogenase.*


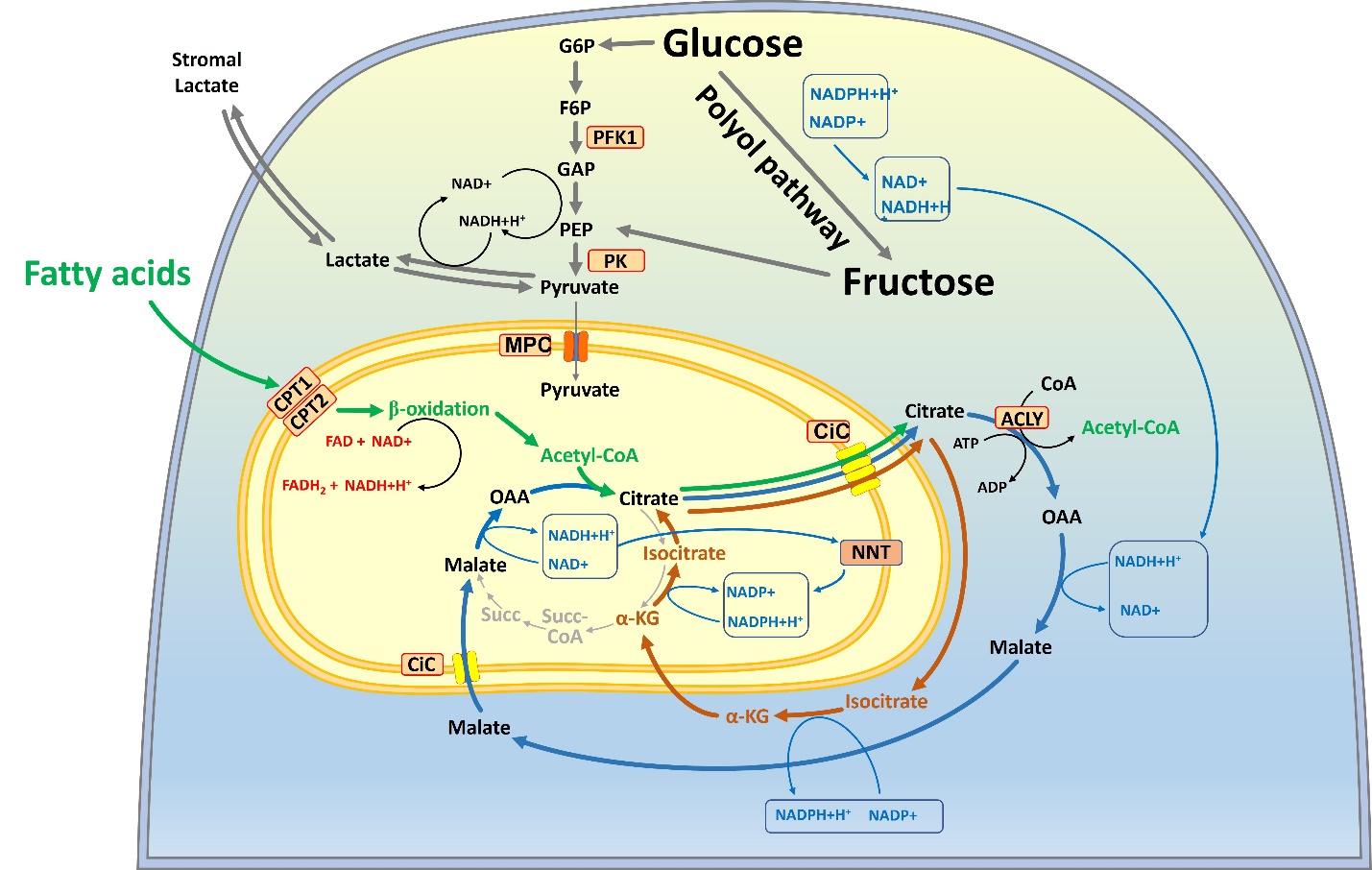


**Figure S3.** Polyol pathway solves the redox equation of the β-oxidation shuttle. The green arrows indicate the metabolic flux from fatty acids. The red arrows indicate the metabolic flux from glucose. The blue arrows indicate the malate-citrate shuttle. The brown arrows indicate the citrate-isocitrate shuttle. *Legend: ACLY – ATP citrate lyase; CIC – mitochondrial citrate carrier; CTP1 and CTP2 – carnitine palmitoyl transferases 1 and 2; F6P – fructose-6-phosphate/fructose-1,6-bisphosphate; G6P – glucose-6-phosphate; GAP – glyceraldehyde-3-phosphate; a-KG – a-ketoglutarate; MPC – mitochondrial pyruvate carrier; NNT – NAD(P) transhydrogenase; OAA – oxaloacetate; PC – pyruvate carboxylase; PFK1 – phosphofructokinase-1; PK – pyruvate kinase; PEP – phosphoenolpyruvate; Succ – succinate.*

**List of all abbreviations**

ACAD – acyl-CoA dehydrogenase

ACAT1 – acetyl-CoA acetyltransferase 1

ACC1 and ACC2 – acetyl-CoA carboxylases 1 and 2

ACLY – ATP-dependent citrate lyase

mACN – mitochondrial aconitase

AK – adenylate kinase

ALDH – aldehyde dehydrogenase

AML – acute myeloid leukemia

AMPK – adenosine monophosphate-activated protein kinase

ATGL – adipose triglyceride lipase

BCL-2 – B-cell lymphoma 2

CIC – citrate/isocitrate carrier

CPT1 and CPT2 – carnitine palmitoyl transferases 1 and 2

CrAT – carnitine acetyltransferase

EH – enoyl-CoA hydratase

ERR-α – estrogen-related receptor alpha

ETC – electron transport chain

ETF – electron transfer flavoprotein

ETF-QO – electron transport flavoprotein-ubiquinone oxidoreductase

FABP – fatty acid binding protein

mFAO – mitochondrial fatty acid oxidation

FAS – fatty acid synthesis

FASN – fatty acid synthase encoded by the FASN gene

FBPase – fructose-1,6-bisphosphatase

FH – fumarate hydratase

HCC – hepatocellular carcinoma

HIF-1α – hypoxia inducible factor one alpha

G6PD – glucose-6-phosphate dehydrogenase

GAPDH – glyceraldehyde 3-phosphate dehydrogenase

HAD – hydroxyacyl-CoA dehydrogenase

ICL – isocitrate lyase

KAT – 3-ketothiolase

α-KG – alpha-ketoglutarate

α-KGDH – alpha-ketoglutarate dehydrogenase

LCAD – long chain acyl-CoA dehydrogenase

LIPT1 – lipoyltransferase 1

MCAD – medium chain acyl-CoA dehydrogenase

MCD – malonyl-CoA decarboxylase

MCL-1 – myeloid leukemia cell differentiated protein 1

MDH1 and MDH2 – malate dehydrogenase 1 and 2

ME1 – malic enzyme 1

MPC – mitochondrial pyruvate carrier

NAD-IDH – NAD-dependent isocitrate dehydrogenase

NFkB – nuclear factor kappa B

NNT – nicotinamide nucleotide transhydrogenase

NRF-2 – nuclear respiratory factor 2

NSCLC – non-small cell lung cancer

OXPHOS – oxidative phosphorylation

PC – pyruvate carboxylase

PDH – pyruvate dehydrogenase

PDK – pyruvate dehydrogenase kinase

PDP – pyruvate dehydrogenase phosphatase

PPP – pentose phosphate pathway

PUFA – polyunsaturated fatty acid

Q and QH2 – coenzyme Q10 (oxidized and reduced forms)

RET – reverse electron transport

ROS – reactive oxygen species

SCAD – short chain acyl-CoA dehydrogenase

SDH – succinate dehydrogenase

SQR – succinate-coenzyme Q reductase

STAT1 – signal transducer and activator of transcription 1

TFP – mitochondrial trifunctional fatty acid oxidation enzyme

VLCAD – very long chain acyl-CoA dehydrogenase

YAP yes-associated protein
